# Jagged/Notch proteins promote endothelial‐mesenchymal transition‐mediated pulmonary arterial hypertension via upregulation of the expression of GATAs

**DOI:** 10.1111/jcmm.17723

**Published:** 2023-03-20

**Authors:** Kun‐Chen Lin, Jui‐Ning Yeh, Pei‐Lin Shao, John Y. Chiang, Pei‐Hsun Sung, Chi‐Ruei Huang, Yi‐Ling Chen, Hon‐Kan Yip, Jun Guo

**Affiliations:** ^1^ Department of Anesthesiology Kaohsiung Chang Gung Memorial Hospital and Chang Gung University College of Medicine Kaohsiung Taiwan; ^2^ Department of Cardiology The First Affiliated Hospital of Jinan University Guangzhou China; ^3^ Department of Nursing Asia University Kaohsiung Taiwan; ^4^ Department of Computer Science and Engineering National Sun Yat‐Sen University Kaohsiung Taiwan; ^5^ Department of Healthcare Administration and Medical Informatics Kaohsiung Medical University Kaohsiung Taiwan; ^6^ Division of Cardiology, Department of Internal Medicine Kaohsiung Chang Gung Memorial Hospital and Chang Gung University College of Medicine Kaohsiung Taiwan; ^7^ Center for Shockwave Medicine and Tissue Engineering Kaohsiung Chang Gung Memorial Hospital Kaohsiung Taiwan; ^8^ Institute for Translational Research in Biomedicine Kaohsiung Chang Gung Memorial Hospital Kaohsiung Taiwan; ^9^ Department of Medical Research, China Medical University Hospital China Medical University Taichung Taiwan; ^10^ Division of Cardiology, Department of Internal Medicine Xiamen Chang Gung Hospital Xiamen China

**Keywords:** endothelial‐mesenchymal transition biomarkers, intercellular signalling, propylthiouracil, pulmonary artery hypertension, vascular wall proliferation

## Abstract

This study tested the hypothesis that Jagged2/Notches promoted the endothelial‐mesenchymal transition (endMT)‐mediated pulmonary arterial hypertension (PAH) (i.e. induction by monocrotaline [MCT]/63 mg/kg/subcutaneous injection) through increasing the expression of GATA‐binding factors which were inhibited by propylthiouracil (PTU) (i.e. 0.1% in water for daily drinking since Day 5 after PAH induction) in rodent. As compared with the control (i.e. HUVECs), the protein expressions of GATAs (3/4/6) and endMT markers (Snail/Zeb1/N‐cadherin/vimentin/fibronectin/α‐SMA/p‐Smad2) were significantly reduced, whereas the endothelial‐phenotype markers (CD31/E‐cadherin) were significantly increased in silenced *JAG2* gene or in silenced *GATA3* gene of HUVECs (all *p* < 0.001). As compared with the control, the protein expressions of intercellular signallings (GATAs [3/4/6], Jagged1/2, notch1/2 and Snail/Zeb1/N‐cadherin/vimentin/fibronectin/α‐SMA/p‐Smad2) were significantly upregulated in TGF‐ß/monocrotaline‐treated HUVECs that were significantly reversed by PTU treatment (all *p* < 0.001). By Day 42, the results of animal study demonstrated that the right‐ventricular systolic‐blood‐pressure (RVSBP), RV weight (RVW) and lung injury/fibrotic scores were significantly increased in MCT group than sham‐control (SC) that were reversed in MCT + PTU groups, whereas arterial oxygen saturation (%) and vasorelaxation/nitric oxide production of PA exhibited an opposite pattern of RVW among the groups (all *p* < 0.0001). The protein expressions of hypertrophic (ß‐MHC)/pressure‐overload (BNP)/oxidative‐stress (NOX‐1/NOX‐2) biomarkers in RV and the protein expressions of intercellular signalling (GATAs3/4/6, Jagged1/2, notch1/2) and endMT markers (Snail/Zeb1/N‐cadherin/vimentin/fibronectin/TGF‐ß/α‐SMA/p‐Smad2) in lung parenchyma displayed an identical pattern of RVW among the groups (all *p* < 0.0001). Jagged‐Notch‐GATAs signalling, endMT markers and RVSBP that were increased in PAH were suppressed by PTU.

## 1 BACKGROUND

Histopathological features of pulmonary vasculatures in idiopathic pulmonary arterial hypertension (PAH) have been clearly identified as complicated morphological changes, including vasoconstriction of resistance artery, in situ thrombosis in small arteries and pulmonary vascular remodelling by proliferation of smooth muscle cells along with intimal fibrosis, medial hypertrophy and adventitial thickening.^1^, ^2^, ^3^, ^4^, ^5^, ^6^ This, ultimately, causes not only an overproduction of vasoconstrictors such as endothelin‐1 and tissue proliferative factors but also a concomitant reduction in vasodilators and antiproliferative factors, such as prostacyclin and nitric oxide.^7^ Currently, there still lacking the effective treatment for PAH, raising that the scientists, in addition to developing a new strategic management for this unmet need, should clearly delineate the underlying mechanism of PAH for a precise treatment.

Activation of *JAG2* (i.e. gene expression) in breast tumour cells induced epithelial mesenchymal transition (EMT) and promoted cell survival and migration has been well recognized by previous study.^8^ Interestingly, a study has previously demonstrated that depleted *JAG2* gene intensely inhibited pancreatic cancer cell migration, invasion and metastasis.^9^ Additionally, Jagged2 (i.e. protein expression) was also identified to be remarkably increased in bone marrow stromal cells under hypoxia and promoted the self‐renewal of cancer stem‐like cells by activating Notch signalling.^10^ Presently, Notch activation is identified to be almost always dependent upon Jagged2 ligand engagement.^11^ Moreover, a previous study has demonstrated that expression of Notch ligand of Jagged2 was closely correlated with different grades of metastatic and recurrent bladder carcinoma.^12^ This finding^12^ suggested that Jagged2 plays an important signalling role in tumour metastasis in bladder cancer through initiation of EMT process.

Of the GATA family,^13^, ^14^, ^15^, ^16^ the GATA3 is the most critical regulator of mammary epithelial cells.^17^ Additionally, transcription factors of the GATA family are essential regulators of the specification, differentiation and proliferation of numerous tissues and a broad variety of cancers in humans, including leukaemia, breast cancer, gastrointestinal cancers and others.^13^, ^14^, ^15^, ^16^

The endMT is a process in which endothelial cells (ECs) lose polarity and cell‐to‐cell contacts, and undergo a dramatic remodelling of the cytoskeleton, called cellular trans‐differentiation.^18^, ^19^, ^20^ The endMT is a complex biological process in which ECs adopt a mesenchymal phenotype displaying typical mesenchymal cell morphology and functions, including the acquisition of cellular motility and contractile properties.^18^, ^19^, ^20^, ^21^, ^22^ Studies have further established that the ECs undergoing endMT lose the expression of epithelial cell‐specific proteins and initiate the expression of mesenchymal cell‐specific genes as well as the production of their encoded proteins.^23^

Intriguingly, the process of EMT/endMT is regulated by several transcriptional suppressor families, including the zinc‐finger proteins (i.e. Zeb 1 and Zeb 2), Snail1 and Snail2.^24^, ^25^, ^26^ Zeb1 and Zeb2 expressions are regulated by the microRNA‐200 (i.e. called family miR‐200).^27^, ^28^, ^29^ Furthermore, a previous study has shown upregulation of miR‐200 locks the tumour cells into an epithelial state and abrogates their metastatic capacity.^30^ Moreover, one study has exhibited that Jagged2 was found to promote metastasis of mouse lung adenocarcinoma cells by increasing the expression of GATA‐binding factors, which suppressed the expression of miR‐200 family that targeted at the transcriptional repressors that, in turn, drove EMT and thereby induced EMT, suggesting that GATA3 and miR‐200 are mutually inhibitory and have opposing effects on EMT and metastasis.^8^ Intriguingly, studies have further demonstrated that PAH was associated with GATA2/6 deficiency.^31^, ^32^, ^33^ On the hand, studies have shown that GATA4 is upregulated in setting of PAH.^34^

It is well known the pathogenesis PAH shares quite similarities with carcinogenesis. Additionally, some studies have previously demonstrated that (EndMT) played a crucial role in PAH through upregulating the vascular wall remodelling.^21^, ^35^, ^36^ Accordingly, it is rationale to hypothesize that endMT plays a principal role in initiation and progression of PAH. However, the characteristics of endothelial cells which have undergone endMT in PAH have not yet been fully investigated. Some previous studies have shown that activation of Jagged‐Notch signalling enhance pulmonary vascular smooth muscle cell proliferation.^37^, ^38^ However, there have no data to address the role of Jagged2 or GATA3 on setting of PAH. Based on the aforementioned mentioned issues,^18^, ^19^, ^20^ we proposed that a complex signalling of Jaggeds‐Notches‐GATAS‐family miR‐200 might participate in the initiation and propagation of PAH though regulating the endMT process in PA.

The PTU is well‐known for treatment of hyperthyroidism. In addition to its standard clinical use for patients with hyperthyroidism, because of its thyroid‐suppressing function, PTU has also been identified to own a strong antioxidant ability,^39^ augment NO production, inhibit vascular smooth muscle proliferation and migration, as well as collagen production.^40^, ^41^ Accordingly, previous studies,^42^, ^43^ including ours,^44^ have revealed that PTU has a strong anti‐atherosclerotic effect. Furthermore, another of our previous study^45^ has shown that PTU therapy attenuated monocrotaline (MCT)‐induced PAH through augmenting NO production and suppressing smooth muscle cell proliferation. However, the underlying mechanism of PTU therapy on ameliorating PAH is still not yet fully clarified by our previous study.^45^

## 2 MATERIALS AND METHODS

### 2.1 Procedure and protocol of cell culture

Human umbilical vein endothelial cells (HUVECs) (BCRC H‐UV001) at passages 3–6 were cultured in M199 medium (Gibco) that contained 20% Fetal Bovine Serum (Hyclone), 1% ECGS (Millipore), 0.2% Heparin (China Chemical & Pharmaceutical Co., Ltd), 100 μg/ml streptomycin and 100 units/ml penicillin (Gibco). In detail, 1 × 10^6^ HUVEC cells were seeded in a 10‐cm dish. After overnight incubation, cells were treated with 10 ng/ml of transforming growth factor (TGF)‐β1 (R&D), 2 μM of monocrotaline (MCT) (Sigma) or 20 ng/ml propylthiouracil (PTU) (Panbiotic Laboratories Co., Ltd.). After additional incubation for 48 h, the cells were collected, and protein extract was harvested with RIPA buffer for Western blot analysis.

### 2.2 The procedure and protocol of silencing *JAG2* and *GATA3*

Transient transfection of cells with siRNA‐*JAG2* and siRNA‐*GATA3* were performed with Lipofectamine® RNAiMAX Transfection Reagent (Invitrogen, Life Technologies) according to the manufacturer's instructions with minimal modification. Briefly, 1 × 10^6^ HUVEC cells grew in a 10‐cm plastic dish overnight. For the purpose of transfection, 18 μl of Lipofectamine® RNAiMAX Transfection Reagent was incubated with 60 pmoL of siRNA at room temperature for 10 min. After the complex was added to the cells, the cells were incubated at 37°C in a humidified atmosphere of 5% CO_2_ for 24 h. qRT‐PCR technique was performed to evaluate the efficiency of silenced *JAG2* and *GATA3*.

### 2.3 Transwell migration assay

Cells were first trypsinized, and 5 × 10^4^ cells were then added to the Boyden chambers (8 μm pore size; Millipore) with 0.5% FBS‐containing medium and assay media that contained 10% FBS was added to the culture plates. After 24 h incubation, the non‐motile cells at the top of the filter were removed and the motile cells at the bottom of the filter were fixed with methanol and stained with one‐tenth dilution of Giemsa (Sigma Corporation). The number of migrated cells in each chamber was carefully counted in five randomly chosen fields under the microscope for three independent experiments.

### 2.4 Animal model of PAH and animal grouping

It is well recognized that MCT treatment is one of the most popular method for creating a validated animal model of PAH.^45^, ^46^ The procedure and protocol were based on our previous reports.^45^, ^46^ In detail, by Day 0, pathogen‐free, adult male Sprague–Dawley (SD) rats, weighing 325–350 g (Charles River Technology, BioLASCO Co., Ltd., *n* = 24) were given one subcutaneous injection of monocrotaline (MCT) (63 mg/kg; Sigma). On Day 5, these MCT‐treated animals were assigned to two experimental groups: group 2 (MCT alone, *n* = 8) and group 3 (MCT + 0.1% PTU [Sigma] in drinking water daily, *n* = 8). Another group 1 (i.e. sham‐operated control [SC], *n* = 8) that receiving only subcutaneous injection of 3 ml normal saline served as SC. PTU therapy was implemented immediately after the assignment. The dosage of MCT utilized here was based on our previous reports^45^, ^46^ with minimal modification of MCT dosage.

### 2.5 Rationale of the PTU dosage

The dosage and the time point of the PTU administration were according to our previous description.^45^ Briefly, the 0.1% PTU in water 100 ml was equivalent to 0.27 mg per 100 ml of drinking water which was the average daily amount of water consumption for each rat in our study. It was well recognized that a rat usually drank 15–18 cc water per day. Thus, it was easily to calculate the dosage of PTU for one rat per day.

### 2.6 Hemodynamic measurement

The procedure and protocol have been described in our previous studies.^45^, ^46^ In detail, on Day 42 after MCT administration, the rats were anaesthetised by inhalational 2.0% isoflurane, placed supine on a warming pad at 37°C for midline laparotomies. After being shaved on the chest, animals in each group were endotracheally intubated with positive‐pressure ventilation (180 ml/min) with room air using a small animal ventilator (SAR‐830/A, CWE Inc.). The heart was exposed by left thoracotomy. A sterile 20‐gauge, soft plastic‐coated needle was inserted into the right ventricle (RV) and femoral artery of each rat to measure the right ventricular systolic pressure (RVSP) (an indirect indicator of pulmonary arterial systolic blood pressure) and arterial pressure, respectively. The pressure signals were first transmitted to pressure transducers (UFI, model 1050) and were then exported to a bridge amplifier (ML866 PowerLab 4/30 Data Acquisition Systems. ADInstruments Pty Ltd.) where the signals were amplified and digitized. The data were recorded and later analysed with the Labchart software (ADInstruments). After hemodynamic measurements, the rats were euthanized in each group with the hearts and lungs to be harvested for individual study. For each animal, the right ventricular weight, whole heart weight, septal + left ventricular (LV) weight and body weight were recorded. The left lung was fixed in 4% formaldehyde and then embedded in paraffin blocks. The right lung was cut into pieces, frozen in liquid nitrogen and then stored at −80°C until future use.

### 2.7 Measurement of pulmonary arterial contractility

At the end of study, pulmonary artery was isolated, cleaned and cut into slices of 2 mm in length for evaluating the contractile and relaxant response as we previously reported.^47^ In detail, pulmonary arterial rings were carefully mounted on an isometric force transducer (420A, Danish Myo Technology) with a tension of 1.0 g and placed in an organ chamber filled with Krebs solution (NaCl, 99.01 mM; KCl, 4.69 mM; CaCl_2_, 1.87 mM; MgSO_4_, 1.20 mM; K_2_HPO_4_, 1.03 mM; glucose, 11.1 mM) maintained at pH 7.4 and bubbled with 95% O_2_ + 5% CO_2_. After an equilibration of 40 min, 1 μM of phenylephrine (PE) was added to the organ chamber for the assessment of contractile activity, and then, 30 μM of acetylcholine (ACh) was added to assess the endothelial integrity. After washing and a re‐equilibration for 30 min, a cumulative PE dose (from 1 nM to 1 μM) and then sodium nitroprusside (SNP) (30 μM) were added to the organ chamber to obtain a concentration‐dependent contractile curve and a relaxant response, respectively. After washing and a re‐equilibration for 20 min, 30 μM of ACh was added in the organ chamber followed by 1 μM of PE to evaluate the endothelium‐dependent vasorelaxant response. Then, PE (1 μM)‐induced vasocontractile response was assessed again in the presence of L‐NAME (100 μM) pre‐treatment for 30 min. All data were acquired and analysed using the DMT (Danish Myo Technology) dual wire myograph system.

### 2.8 Assessment for the arterial basal nitric oxide (NO) release

Vascular basal nitric oxide release was calculated as the percentage of difference between PE‐induced vasocontractile response in the absence and presence of L‐NAME according to our previous study.^47^

### 2.9 Histological assessment of lung injury and crowded score of lung parenchyma and vascular wall remodelling of pulmonary artery

To elucidate the alveolar sac distribution in lung parenchyma, left lung specimens from all animals were fixed in 10% buffered formalin before embedding in paraffin and the tissue was sectioned at 5 μm for light microscopic analysis. The H&E staining was performed to determine the number of alveolar sacs according to our previous study^48^ in a blind fashion. Three lung sections from each rat were analysed, and three randomly selected high‐power fields (HPFs) (200×) were examined in each section. The mean number per HPF for each animal was then determined by summation of all numbers divided by 9. In addition, alveolar wall thickness and the presence or absence of haemorrhage were determined under light microscopy. The extent of crowded area, which is defined as region of thickened septa in lung parenchyma associated with partial or complete collapse of alveoli on H&E‐stained sections, was performed in a blind fashion. The scoring system adopted was as follows: 0 = no detectable crowded area; 1 = <15% of crowded area; 2 = 16%–25% of crowded area; 3 = 26%–50% of crowded area; 4 = 51%–75% of crowded area; 5 = 76%–100% of crowded area/per high‐power field (200×). Additionally, this scoring system was also adapted for identification of the lung inflammatory cell infiltration score (i.e. to replace the ‘detectable crowded area’ by ‘inflammatory cell infiltration area’).

The procedure and protocol for assessing the muscularization (i.e. an index of vascular wall remodelling) of pulmonary arterioles have described in detail in our previous report.^45^, ^49^ Briefly, three measurements were examined for the analysis of thickness of pulmonary arterioles. Muscularization of the pulmonary arterial medial layer at the level of middle lung level was defined as a mean thickness of vessel wall >50% of the lumen diameter in a vessel of diameter >30 μm. Measurement of arteriolar diameter and wall thickness was achieved using the Image‐J software (NIH).

In this study, histopathological findings were examined by Olympus BX51 microscope (Shinjuku). The lens used in this study were the Olympus UPlanSApo 40× Plan Apo objective lens (40×/0.90) for 400× magnification and the Olympus UPlanSApo 20× Plan Apo objective lens (20×/0.75) for 200× magnification.

### 2.10 Western blot analysis

Western blot analysis was performed as our previous description.^45^, ^46^, ^47^, ^48^ In detail, equal amounts (50 μg) of protein extracts were loaded and separated by SDS‐PAGE. Separated proteins were transferred to PVDF membranes, and nonspecific sites were blocked by incubation in blocking buffer (5% nonfat dry milk in T‐TBS [TBS containing 0.05% Tween 20]) overnight. The membranes were incubated with the indicated primary antibodies Jagged1 (1:1000, GeneTex), Jagged2 (1:1000, Sigma), Notch1 (1:1000, Abcam), Notch2 (1:1000, Cell Signaling), Snail (1:1000, Cell Signaling), Zeb1(1:1000, Cell Signaling), E‐cadherin (1:1000, Abcam), N‐cadherin (1:1000, Cell Signaling), Fibronectin (1:1000, Abcam), Vimentin (1:1000, Cell Signaling), phosphorylated (p)‐Smad3 (Ser423/425) (1:1000, Cell Signaling), GATA3 (1:1000, Abcam), GATA4 (1:1000, Proteintech), GATA6 (1:1000, Affinity Biosciences), CD31 (1:1000, Abcam), alpha‐smooth muscle actin (α‐SMA) (1:1000, Sigma), p‐Smad2 (Ser465/467) (1:1000, Cell Signaling), brain natriuretic peptide (BNP) (1:1500, Abcam), ß‐myosin heavy chain (ß‐MHC) (1:1000, Santa cruz), α‐MHC (1:1000, Santa cruz), p‐Connexin43 (1:1000, Cell Signaling), TGF‐ß (1:1000, Abcam) and actin (1:10,000, Chemicon) for 1 h at room temperature. Horseradish peroxidase‐conjugated anti‐rabbit immunoglobulin IgG (1:2000, Cell Signaling) was used as a secondary antibody for 1‐h incubation at room temperature. The washing procedure was repeated eight times within 1 h. Immunoreactive bands were visualized by enhanced chemiluminescence (ECL; Amersham Biosciences) and exposed to Biomax L film (Kodak). For purposes of quantification, ECL signals were digitized using Labwork software (UVP).

### 2.11 Immunofluorescent (IF) staining

The procedure and protocol have been described in our previous reports.^45^, ^46^, ^47^, ^48^ Re‐hydrated paraffin sections were treated with 3% H_2_O_2_ for 30 min and incubated with Immuno‐Block reagent (BioSB, Santa Barbara, CA, USA) for 30 min at room temperature. Sections were then incubated with primary antibodies against GR (1:400, Abcam), GPX (1:300, Abcam), CD68 (1:500, Abcam) and CD11b (1:100, Abcam). Three sections of lung specimens from each rat were analysed. For quantification, three randomly selected HPFs (200× for IHC and IF studies) were analysed in each section. The mean number of positively stained cells per HPF for each animal was determined by summation of all numbers divided by 9.

### 2.12 Histological quantification of lung fibrosis

The procedure and protocol have been described in our previous reports.^44^, ^45^, ^46^, ^47^, ^48^ In detail, Masson's trichrome staining was used for identification of the lung fibrotic area. Three serial sections of lung in each animal were prepared at 4 μm thickness by Cryostat (Leica CM3050S). The integrated areas (μm^2^) of fibrosis on each section were calculated using the Image Tool 3 (IT3) image analysis software (University of Texas, Health Science Center, San Antonio, UTHSCSA; Image Tool for Windows, Version 3.0). Three randomly selected high‐power fields (HPFs) (100×) were analysed in each section. After determining the number of pixels in each fibrotic area per HPF, the numbers of pixels obtained from three HPFs were calculated. The procedure was repeated in two other sections for each animal. The mean pixel number per HPF for each animal was then determined by summing up all pixel numbers and divided by 9. The mean integrated area (μm^2^) of fibrosis in parenchyma per HPF was obtained using a conversion factor of 19.24 (since 1 μm^2^ corresponds to 19.24 pixels).

### 2.13 Statistical analysis

Quantitative data were expressed as mean ± standard error of the mean (SEM). Statistical analysis was adequately performed by anova followed by Bonferroni multiple comparison post hoc test. Statistical analysis was performed using SAS statistical software for Windows version 8.2 (SAS Institute). A probability value <0.05 was considered statistically significant.

## 3 RESULTS

### 3.1 TGF‐ß1 upregulated endothelial‐mesenchymal transition (endMT) process

First, to prove that the TGF‐ß1 participated in endMT processing, we categorized the HUVECs into HUVECs only (i.e. the control group) and HUVECs +10 ng/ml of TGF‐ß1 for two time points (i.e. cell culture for 72 h and 120 h, respectively) and utilized the Western blot analysis (Figure S1). The result showed that as compared with control group, the protein expressions of smooth muscle 22 alpha (SM22α), Snail, vimentin, fibronectin and p‐Samd2, five indicators of endMT biomarkers, were markedly upregulated at time point of 72 h and more upregulated at time point of 120 h undergoing the TGF‐ß1 treatment, whereas the protein expression of CD31, an indicator of endothelial phenotype, displayed an opposite pattern of Snail among the groups, suggesting that TGF‐ß1 involved in endMT process.

### 3.2 Protein expressions of endMT biomarkers undergoing the TGF‐ß1 and MCT treatments

Second, for the purpose of MCT would be utilized for PAH induction in rats, we compared whether the MCT treatment was comparable to TGF‐ß1 on upregulating the endMT biomarkers (Figure S2). Accordingly, the HUVECs were categorized into three groups: group I (HUVECs only), group II (HUVECs +10 ng/ml of TGF‐ß1 for 72 h cell culture) and group III (HUVECs +2 μM of MCT), and Western blot analysis was utilized again. The result demonstrated that the protein expressions of Snail, N‐Cadherin, fibronectin, Vimentin, Zeb1 and p‐Smad3, five endMT biomarkers, were notably increased in group II and more notably increased in group III than in group I. Additionally, the protein expressions of Jagged2 and Notch1, two endMT processing up‐regulators (i.e. promoters), exhibited an identical pattern of Snail, whereas the protein expression E‐Cadherin, an index of endothelial phenotype, displayed an opposite pattern of Snail among the groups, suggesting that MCT and TGF‐ß augment the propagation of endMT processing.

### 3.3 Jagged2 regulated the protein expression of GATAs

**FIGURE 1:**
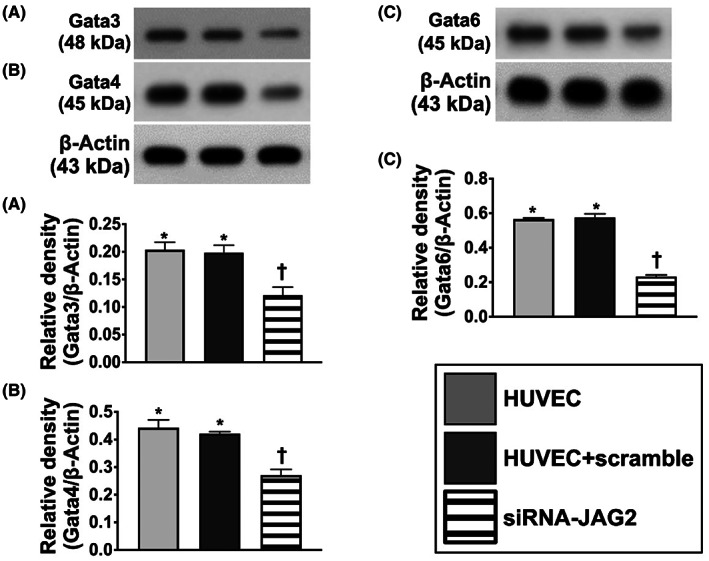
Jagged2 regulated the protein expression of GATAs. (A) Protein expression of GATA3, * versus †, *p* < 0.01. (B) Protein expression of GATA4, * versus †, *p* < 0.01. (C) Protein expression of GATA6, * versus †, *p* < 0.01. Statistical analysis by unpaired Student *t*‐test (*n* = 3 in each group). HUVECs, human umbilical vein endothelial cells; siRNA‐*JAG2*, silenced the *JAG2* gene.

To elucidate whether the Jagged2 regulated the protein expression of GATAs, the gene *JAG2* was silenced in HUVECs and the culturing cells were categorized into HUVECs only, HUVECs + scramble and siRNA‐*JAG2* (Figure 1 and Figure S3). The result showed that the protein expressions of GATA3, GATA4 and GATA6 were significantly lower in siRNA‐*JAG2* group than in the former two groups but they did not differ between the former two groups, suggesting the expressions of GATAs were regulated by jagged2 (i.e. served as the upstream signalling mediated the downstream signalling of GATAs).

### 3.4 GATAs regulated expressions of the EMT biomarkers

**FIGURE 2:**
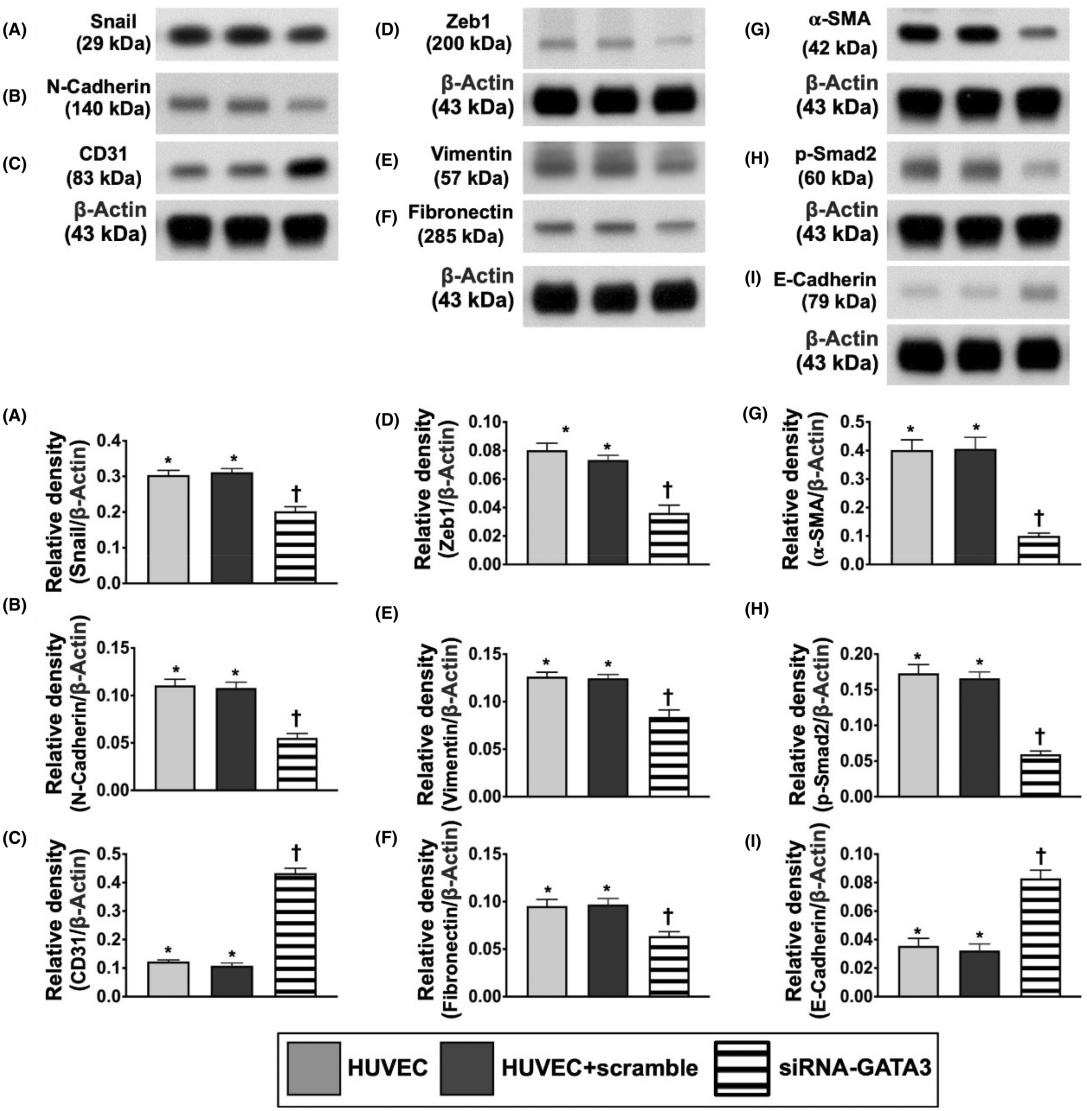
GATAs regulated expressions of the endMT markers. (A) Protein expression of Snail, * versus †, *p* < 0.01. (B) Protein expression of N‐cadherin, * versus †, *p* < 0.01. (C) Protein expression of CD31, * versus †, *p* < 0.01. (D) Protein expression of Zeb1, * versus †, *p* < 0.01. (E) Protein expression of vimentin, * versus †, *p* < 0.01. (F) Protein expression of fibronectin, * versus †, *p* < 0.01. (G) Protein expression of alpha smooth actin (α‐SMA), * versus †, *p* < 0.01. (H) Protein expression of phosphorylated (p)‐Smad2, * versus †, *p* < 0.01. (I) Protein expression of E‐cadherin, * versus †, *p* < 0.01. Statistical analysis by unpaired Student *t*‐test (*n* = 3 in each group). endMT, endothelial‐mesenchymal transition; HUVECs, human umbilical vein endothelial cells; siRNA‐*GATA3*, silenced the *GATA3* gene.

To verify whether the GATAs regulated the expressions of endMT, the silencing of the *GATA3* gene in HUVECs was performed (Figure 2). The result showed that the protein expressions of Snail, Zeb1, N‐cadherin, vimentin, fibronectin, α‐SMA and phosphorylated (p)‐Smad2, six indices of endMT biomarkers, were significantly reduced in siRNA‐*GATA3* group than in HUVECs only and HUVECs + scramble, whereas the protein expressions of CD31 and E‐cadherin, two indicators of the epithelial phenotype biomarkers, exhibited an opposite pattern of Snail among the groups. However, they were similar between HUVECs only and HUVECs + scramble groups. Our findings implicated that the role of the GATAs was essential for augmenting the expressions of EMT biomarkers.

### 3.5 HUVECs migratory assay, miR‐200 expression and cell viability in HUVECs

**FIGURE 3:**
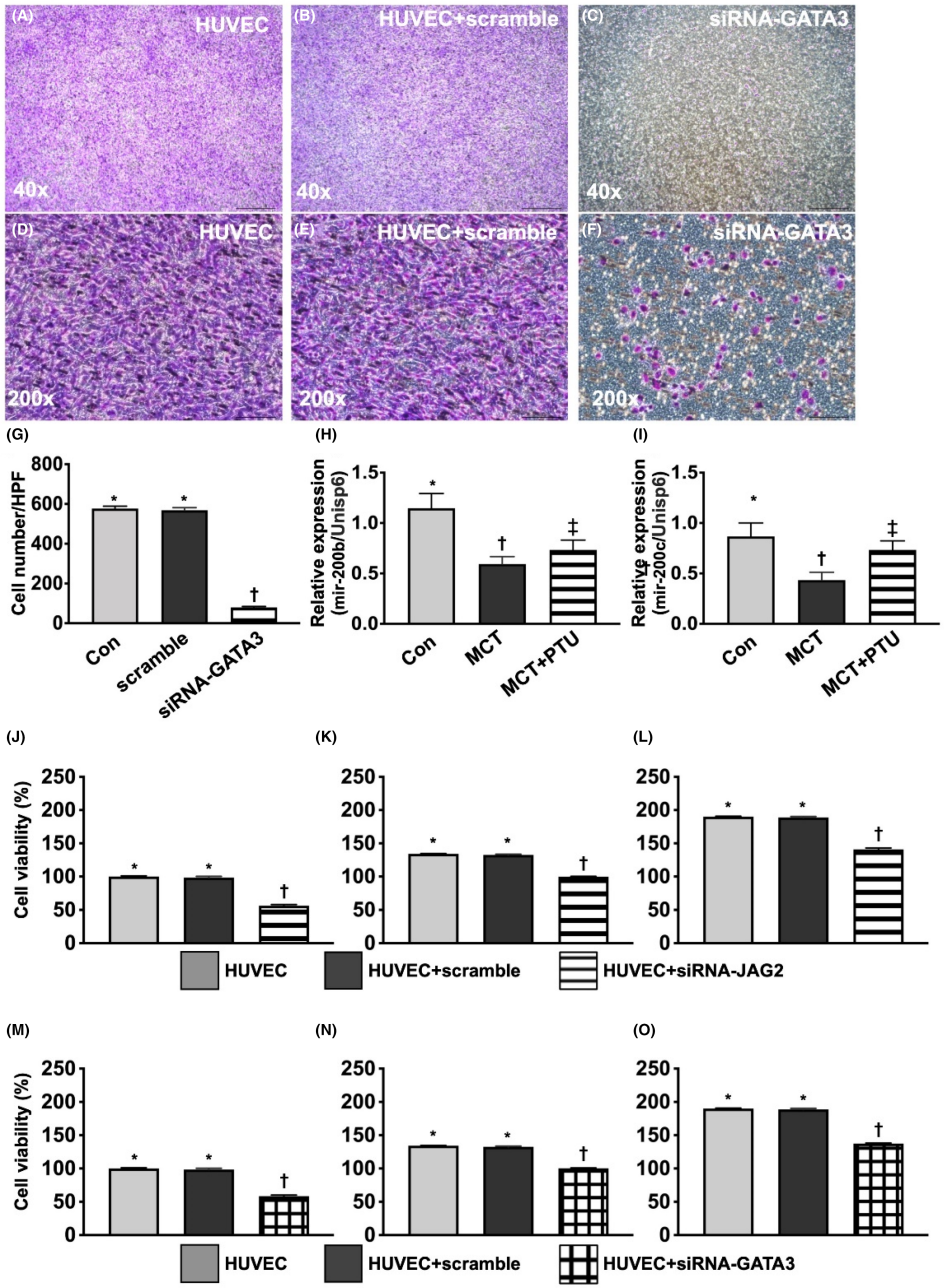
Th capacity of HUVECs migratory assay in condition of siRNA‐*GATA3*. (A–F) Illustrating the microscopic finding of Giemsa stain in low‐power field (i.e. 40×) (A–C) and higher‐power filed (i.e. 200×, scale bars in right lower corners represent 50 μm) (D–F) for identification of the losing migratory ability of HUVECs, that is a reflection of endothelial to mesenchymal processing (i.e. reendothelialization). (G) Analytical result of number of migratory cells, * versus †, *p* < 0.001. Statistical analysis by unpaired Student *t*‐test (*n* = 5 in each group). (H) Relative gene expression of miRNA‐200b‐3p, * versus other groups with different symbols (†, ‡), *p* < 0.01. *n* = 3 for each group. (I) Relative gene expression of miRNA‐200c‐3p, * versus other groups with different symbols (†, ‡), *p* < 0.01. *n* = 3 for each group. (J) Cell viability of Jagged2 at 24 h, * versus †, *p* < 0.001. Statistical analysis by unpaired Student *t*‐test (*n* = 6 in each group). (K) Cell viability of Jagged2 at 48 h, * versus †, *p* < 0.001. Statistical analysis by unpaired Student *t*‐test (*n* = 6 in each group). (L) Cell viability of Jagged2 at 72 h, * versus †, *p* < 0.001. Statistical analysis by unpaired Student *t*‐test (*n* = 6 in each group). (M) cell viability of GATA3 at 24 h, * versus †, *p* < 0.001. Statistical analysis by unpaired Student *t*‐test (*n* = 6 in each group). (N) Cell viability of GATA3 at 48 h, * versus †, *p* < 0.001. Statistical analysis by unpaired Student *t*‐test (*n* = 6 in each group). (O) Cell viability of GATA3 at 72 h, * versus †, *p* < 0.001. Statistical analysis by unpaired Student *t*‐test (*n* = 6 in each group). Other statistical analyses were performed by one‐way anova, followed by Bonferroni multiple comparison post hoc test. Symbols (*, †, ‡) indicate significance (at 0.05 level), this means that * versus †, *p* < 0.05; * versus ‡, *p* < 0.05; † versus ‡, *p* < 0.05 and * versus † versus ‡, *p* < 0.01. HUVECs, human umbilical vein endothelial cells; MCT, monocrotaline; PTU, propylthiouracil; siRNA‐*GATA3*, silenced the *GATA3* gene.

To verify whether the GATAs participated in regulating the propagation of endMT processing, resulting in enhancement of migratory capacity, the HUVECs (2.0 × 10^5^/well were seeded) were categorized into HUVECs only, HUVECs + scramble and silenced *GATA3* gene in HUVECs (i.e. siRNA‐*GATA3*) migratory assay (Figure 3). All the cell cultures were carried for 72 h prior to the cell were collected for individual study. As we expected, the migratory ability was significantly suppressed in siRNA‐*GATA3* group than in the former two groups, but it did not differ between the former two groups. This result proved that in situation of intact of cell viability, losing migratory ability of HUVECs implicated a reflection of endEMT processing. Accordingly, our finding might imply that GATAs participated in upregulating the endothelial to mesenchymal processing (i.e. deendothelialization).

To verify the gene expressions of micro‐RNA 200 family (i.e. miR‐200 family: 200b‐3p, 200c‐3p), the RT‐PCR was performed, the result demonstrated that these two micro‐RNAs were significantly lower in HUVECs + MCT group than in HUVECs and were reversed in HUVECs + MCT group after receiving the PTU treatment. Our finding MCT treatment could attenuated the expressions of micro‐RNA 200 family that was reversed by PTU therapy.

Furthermore, to elucidate the cell viability after silencing the genes of *JAG2* and *GATA3*, the MTT assay was performed and the cells were categorized into HUVECs, HUVEC + Scramble, HUVEC + siRNA‐*JAG2* and HUVEC + siRNA‐*GATA3*, respectively. The result revealed that as compared with control groups, the cell viability was significantly reduced in HUVEC + siRNA‐*JAG2* and HUVEC + siRNA‐*GATA3* at time points of 24, 48 and 72 h.

### 3.6 The therapeutic impact of TGF‐ß1, MCT and PTU on regulating the endMT markers in in vitro study

**FIGURE 4:**
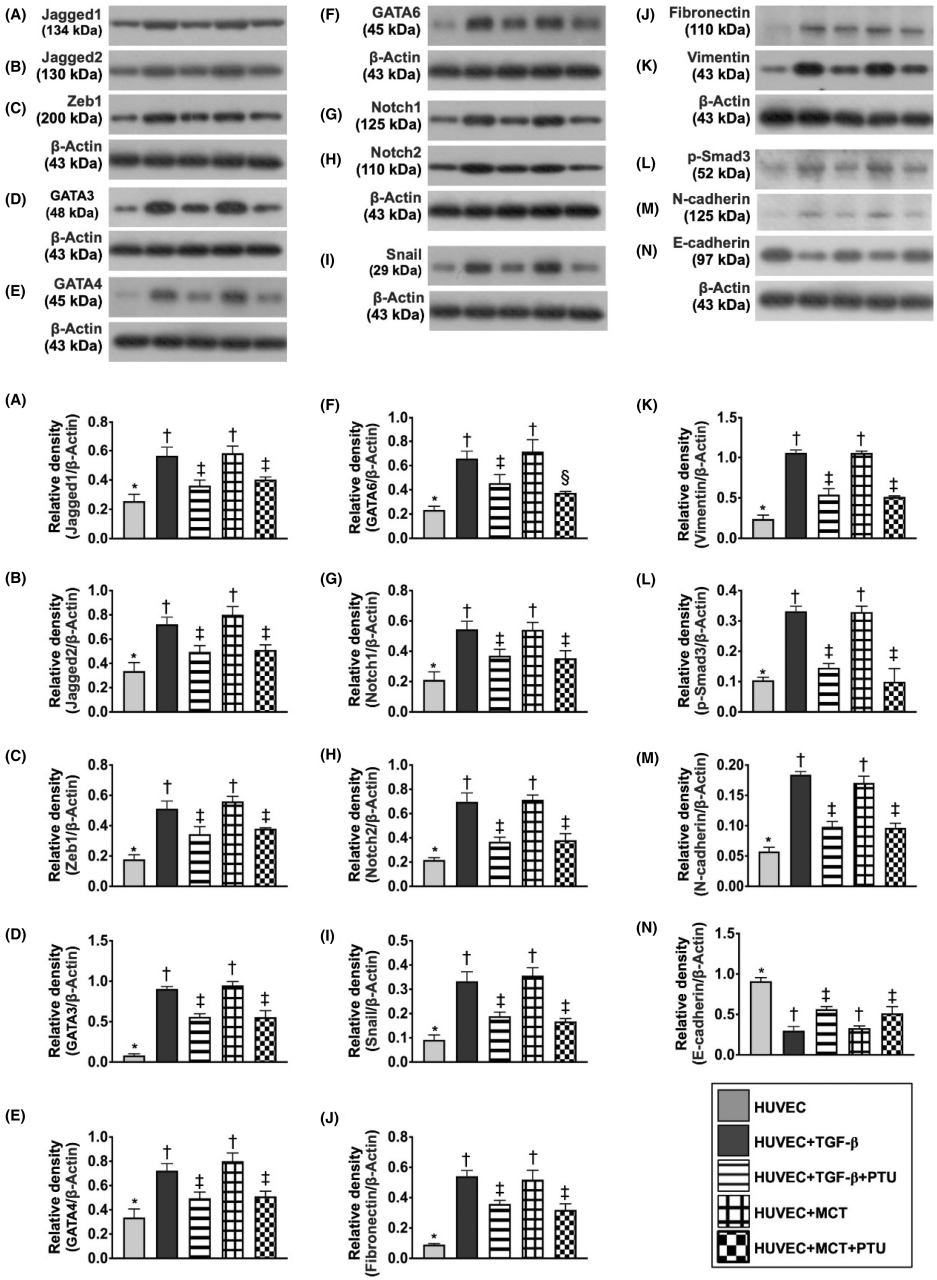
Effect of TGF‐ß1, MCT and PTU on regulating the expression of endMT markers in in vitro study. (A) Protein expression of Jagged1, * versus other groups with different symbols (†, ‡), *p* < 0.001. (B) Protein expression of Jagged2, * versus other groups with different symbols (†, ‡), *p* < 0.001. (C) Protein expression of Zeb1, * versus other groups with different symbols (†, ‡), *p* < 0.001. (D) Protein expression of GATA3, * versus other groups with different symbols (†, ‡), *p* < 0.001. (E) Protein expression of GATA4, * versus other groups with different symbols (†, ‡, §), *p* < 0.001. (F) Protein expression of GATA6, * versus other groups with different symbols (†, ‡), *p* < 0.001. (G) Protein expression of Notch1, * versus other groups with different symbols (†, ‡), *p* < 0.001. (H) Protein expression of Notch2, * versus other groups with different symbols (†, ‡), *p* < 0.001. (I) Protein expression of Snail, * versus other groups with different symbols (†, ‡), *p* < 0.001. (J) Protein expression of fibronectin, * versus other groups with different symbols (†, ‡), *p* < 0.001. (K) Protein expression of Vimentin, * versus other groups with different symbols (†, ‡), *p* < 0.001. (L) Protein expression of phosphorylated (p)‐Smad3, * versus other groups with different symbols (†, ‡), *p* < 0.001. (M) Protein expression of N‐cadherin, * versus other groups with different symbols (†, ‡), *p* < 0.001. (N) Protein expression of E‐cadherin, * versus other groups with different symbols (†, ‡, §), *p* < 0.001. All statistical analyses were performed by one‐way anova, followed by Bonferroni multiple comparison post hoc test (*n* = 3 for each group). Symbols (*, †, ‡, §) indicate significance (at 0.05 level). HUVECs, human umbilical vein endothelial cells; MCT, monocrotaline; PTU, propylthiouracil; TGF‐ß, transforming growth factor beta.

HUVECs were utilized again for assessment of the therapeutic impact of TGF‐ß1 (10 ng/ml), MCT (2 μM) and PTU (20 ng/ml) on the expression of endMT and categorized into five groups: group 1 (HUVECs only), group 2 (HUVECs + TGF‐ß1), group 3 (HUVECs + TGF‐ß1 + PTU), group 4 (HUVECs + MCT) and group 5 (HUVECs + MCT + PTU) (Figure 4). The result demonstrated that the protein expressions of jagged1, jagged2, GATAs (3, 4, 6), Notch1, Notch2, Snail, Zeb1, N‐cadherin, fibronectin, Vimentin, p‐Smad3, 13 indicators of endMT markers, were significantly higher in groups 2 and 4 than in other groups and significantly higher in groups 3 and 5 than in group 1, but they were similar between groups 2 and 4 or between groups 3 and 5, whereas the protein expression of E‐cadherin displayed an opposite pattern of N‐cadherin among the groups, suggesting that PTU therapy effectively suppressed TGF‐ß1 or MCT which were induced by the upregulation of endMT markers.

### 3.7 Pulmonary artery relaxation and nitric oxide (NO) release by Day 42 after MCT treatment

**FIGURE 5:**
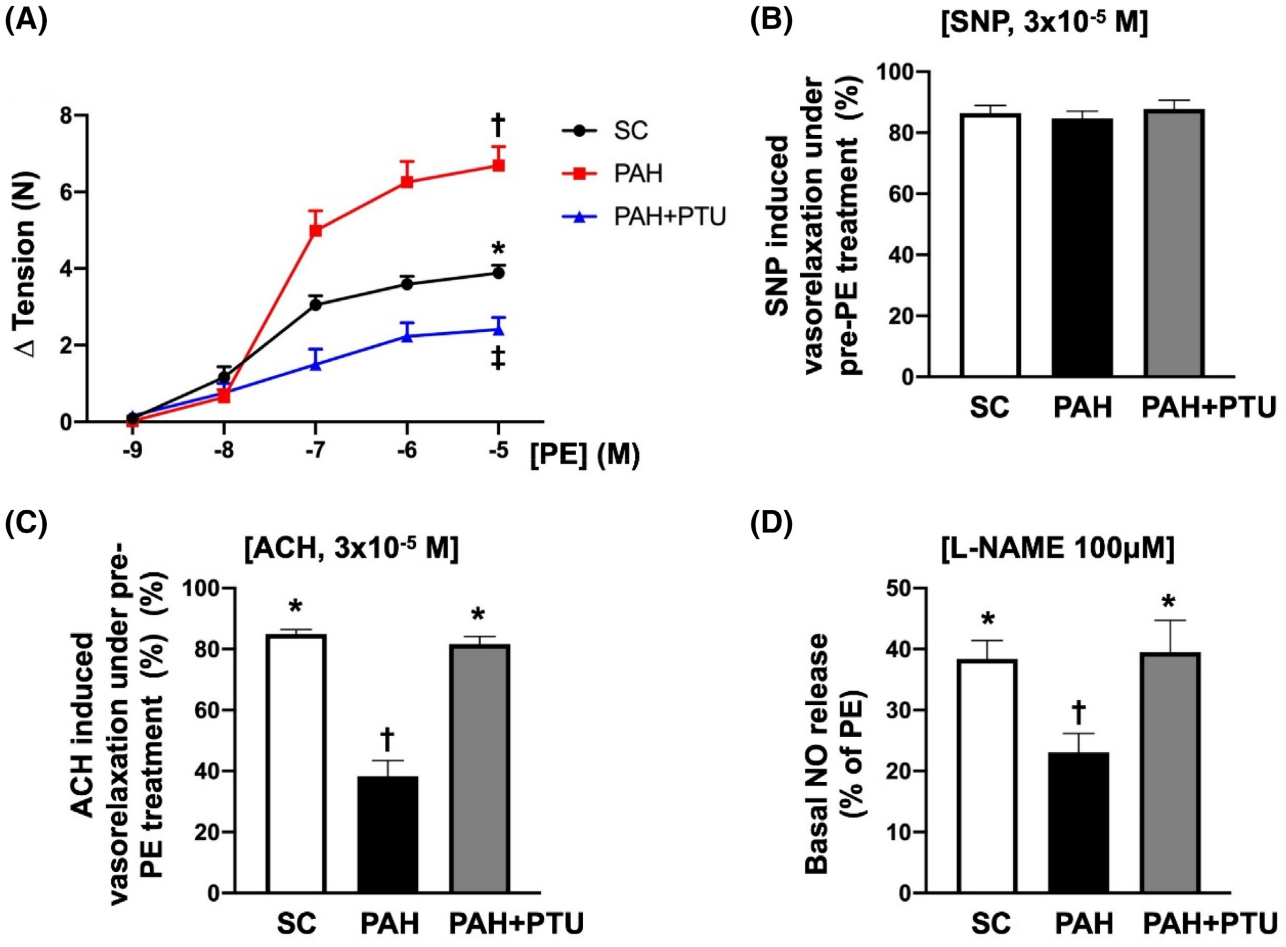
Exo vivo study of pulmonary arterial vasorelaxation and vasoconstriction and nitric oxide (NO) release by Day 42 after MCT treatment. (A) Phenylephrine (PE)‐induced vasoconstriction was significantly increased in MCT group than in other groups. Analytical result of vasoconstriction (%), * versus other groups with different symbols (†, ‡), *p* < 0.0001. (B) Sodium nitroprusside (SNP)‐induced vasorelaxation did not differ among the three groups, suggesting that SNP only acted on the capillaries, that is a direct NO provider. Analytical result of vasorelaxation, *p* > 0.5. (C) Acetylcholine (ACh)‐induced vasorelaxation (i.e. dilatation response) was significantly reduced in MCT group than in the other two groups. Analytical result of vasorelaxation, * versus †, *p* < 0.0001. (D) The baseline NO release from endothelial cells of PA detected by indirect measurement was significantly lower in NCT group than in the other two groups. Analytical result of NO release (%) among the three groups, * versus †, *p* < 0.001. All statistical analyses were performed by one‐way anova, followed by Bonferroni multiple comparison post hoc test (*n* = 8 for each group). Symbols (*, †, ‡) indicate significance (at 0.05 level). MCT, monocrotaline; PTU, propylthiouracil.

The baseline NO released from endothelial cells of PA was significantly reduced in MCT group than in SC that was significantly reversed in MCT‐PTU group (Figure 5).

Additionally, to elucidate the impact of PTU on regulating vessel relaxation and contraction, PA was cut into pieces and mounted on the DMT (Danish Myo Technology). As expected, vasoconstriction induced by phenylephrine was significantly increased in MCT group than in SC and was significantly reversed in MCT‐PTU group. On the contrary, vasorelaxation caused by acetylcholine exhibited an opposite pattern of vasoconstriction among the three groups. Nevertheless, the vasorelaxation caused by sodium nitroprusside did not differ among the groups. Our results supported that PTU therapy could upregulate the PA relaxation and NO production.

### 3.8 Arterial oxygen saturation (SaO_2_ ) (%), right ventricular systolic pressure (RVSBP), LVSBP, femoral arterial systolic blood pressure (FASBP), the ratio of RV weight, whole wet lung weight (WWLW) and septum + LV weight to the tibial length by Day 42 after MCT treatment

**FIGURE 6:**
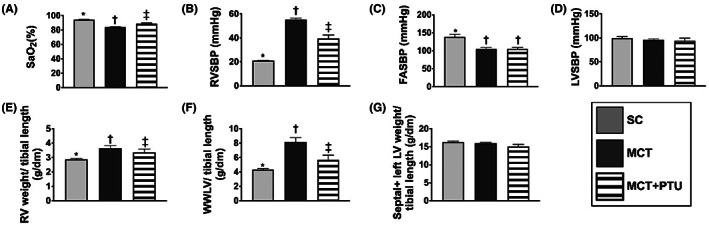
Arterial oxygen saturation (SaO_2_), right ventricular systolic pressure (RVSP), LVSBP, femoral arterial systolic blood pressure (FASBP) and the ratio of RV weight, whole wet lung weight (WWLW) and septum + LV weight to tibial length by Day 42 after MCT treatment. (A) The level of SaO_2_, * versus other groups with different symbols (†, ‡), *p* < 0.001. (B) The value of RVSBP, * versus other groups with different symbols (†, ‡), *p* < 0.0001. (C) The FASBP, * versus †, *p* < 0.001. (D) The left ventricular systolic blood pressure (LVSBP), *p* > 0.5. (E) Ratio of RV weight to tibial length, * versus other groups with different symbols (†, ‡), *p* < 0.0001. (F) The ratio of WWLW to tibial length, * versus other groups with different symbols (†, ‡), *p* < 0.001. (G) The ratio of septal + left LV weight to tibial length, *p* > 0.1. All statistical analyses were performed by one‐way anova, followed by Bonferroni multiple comparison post hoc test (*n* = 6 for each group). Symbols (*, †, ‡) indicate significance (at 0.05 level). MCT, monocrotaline; PTU, propylthiouracil.

By Day 42 after MCT treatment, the value of SaO_2_ was significantly lower in MCT group than in SC and MCT‐PTU groups and significantly lower in MCT‐PTU group than in the SC (Figure 6).

The ratio of RV weight and WWLW to tibial length was significantly increased in MCT group than in SC and MCT‐PTU groups, and significantly increased in MCT‐PTU group than in SC group. On the other contrary, the ratio of septum + LV weight to tibial length showed no significant difference among the three groups.

The RVSBP was significantly higher in MCT group than in the other two groups and significantly higher in MCT‐PTU group than in the SC group. On the contrary, the FASBP was significantly higher in SC than in MCT and MCT‐PTU groups, but it showed no difference between the latter two groups. Nevertheless, the LVSBP did not differ among the three groups. Our finding discovered that PTU therapy not only reduced PAH but also reduced cardiac hypertrophy in setting of PAH.

### 3.9 The lung injury score, lung consolidation area, inflammatory cell infiltration and arterial muscularization Day 42 after MCT treatment

**FIGURE 7:**
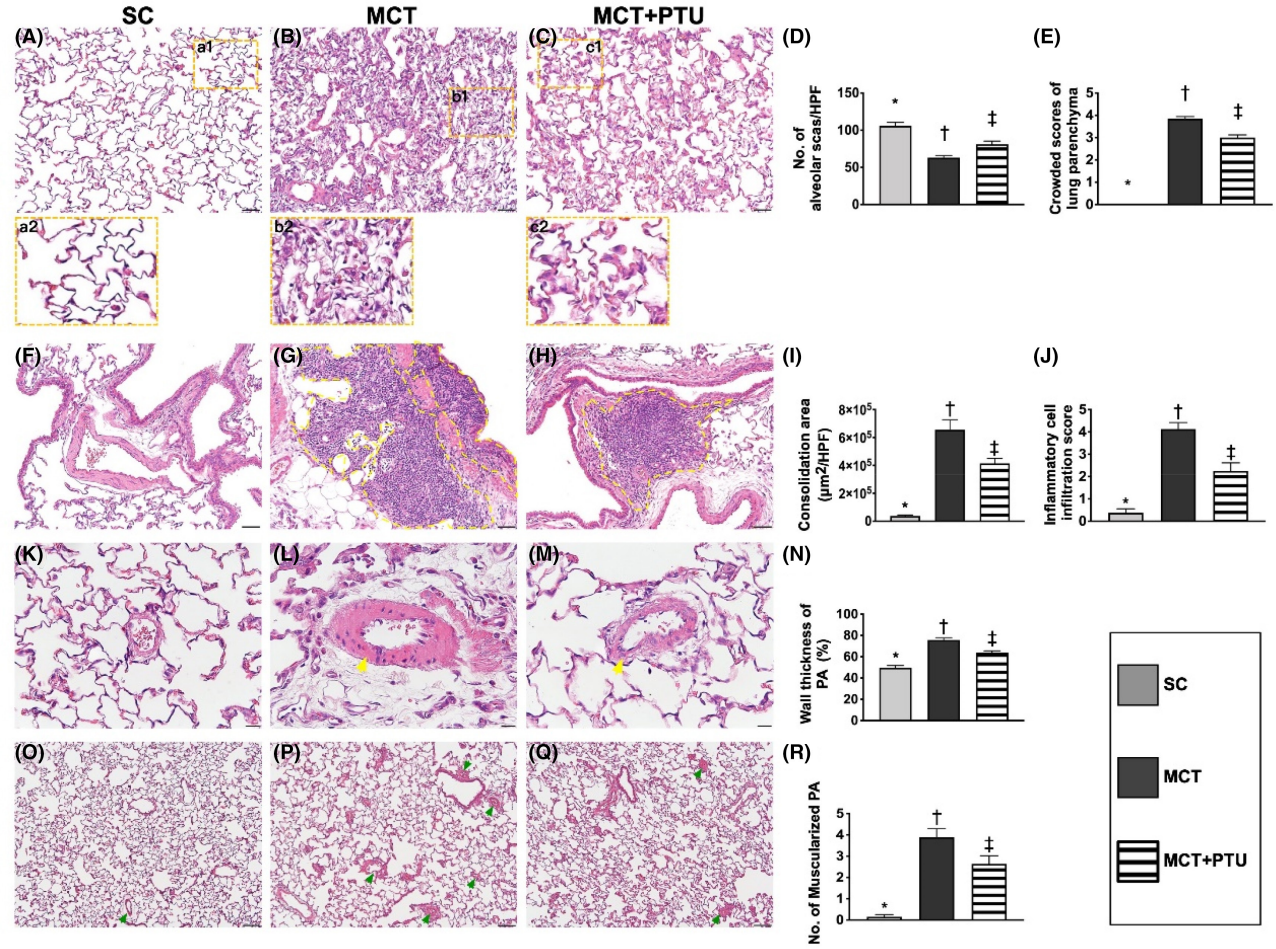
Lung injury score, lung consolidation area and inflammatory cell infiltrating score by Day 42 after MCT treatment. (A–C) Histopathological findings (i.e. H.E. staining) of lung parenchyma under microscopic finding (200×) among the three groups. Small squares of a1, b1 and c1 were magnified to larger squares of a2, b2 and c2, respectively. (D) The number of alveolar sacs among three groups. * versus other groups with different symbols (†, ‡), *p* < 0.0001. (E) Crowded scores of lung parenchyma. * versus other groups with different symbols (†, ‡), *p* < 0.0001. (F–H) Illustrating the microscopic finding (200×) for identification of lung consolidation area (yellow‐dotted line). (I) Analytical result of consolidation area, * versus other groups with different symbols (†, ‡), *p* < 0.0001. (J) Analytical result of inflammatory cell infiltration (grey colour) score, * versus other groups with different symbols (†, ‡), *p* < 0.0001. The scale bars in right lower corners represent 50 μm. (K–M) Illustrating the microscopic finding (400×) of H.E., stain for identification of thickness of pulmonary arterial wall (yellow arrow). The scale bars in right lower corner represent 20 μm. (N) Semi‐quantitative analyses of the wall thickness (%) of PA, * versus other groups with different symbols (†, ‡), *p* < 0.0001. (O–Q) Illustrating the microscopic finding (100×) of H.E., stain for identification of number of muscularized PA (green arrows). The scale bars in right lower corner represent 100 μm. (R) Analytical result of number of muscularized PA, * versus other groups with different symbols (†, ‡), *p* < 0.0001. All statistical analyses were performed by one‐way anova, followed by Bonferroni multiple comparison post hoc test (*n* = 8 for each group). Symbols (*, †, ‡) indicate significance (at 0.05 level). HPF, high‐power filed; MCT, monocrotaline; PTU, propylthiouracil.

To assess the degree of lung injury score and lung consolidation, we utilized the microscopic tool for identification of lung histopathological feature (Figure 7). As we expected, the number of alveolar sacs was significantly reduced in MCT‐PTU group and more significantly reduced in MCT only group as compared with SC group, whereas the lung parenchymal crowding demonstrated an opposite pattern compared to that of the number of alveolar sacs among the three groups, suggesting that the lung injury score (i.e. combined reduction of alveolar sacs and increased crowding lung parenchyma) was notably reversed in PAH animals after receiving the PTU therapy.

Additionally, the lung consolidation area and inflammatory cell infiltration score exhibited an identical pattern of lung injury score among the three groups. Our finding proved that PTU therapy safeguarded the lung architectural integrity in setting of PAH.

Furthermore, histopathological analysis using H&E staining demonstrated that the percentage of thickened arterioles and the number of muscularized arterioles displayed an identical manner compared to that of the crowded score among the three groups.

### 3.10 Fibrotic area and antioxidant biomarker in lung parenchyma by Day 42 after MCT treatment

**FIGURE 8:**
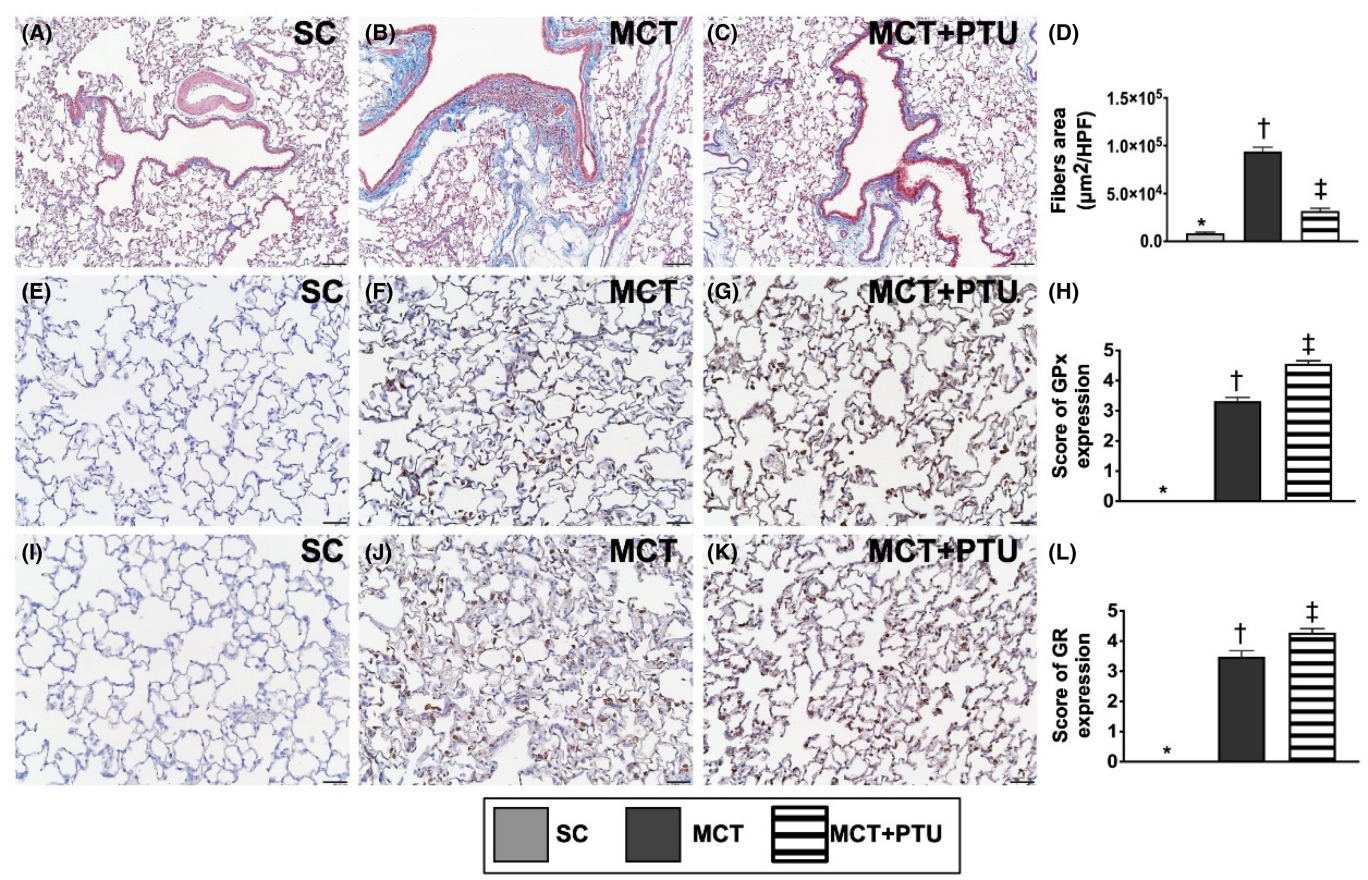
Fibrotic area and antioxidant biomarkers in lung parenchyma by Day 42 after MCT treatment. (A–C) Illustrating the microscopic finding (100×) of Masson's trichome stain for identification of fibrosis in lung fibrosis (blue colour). (D) Analytical result of fibrotic area, * versus other groups with different symbols (†, ‡), *p* < 0.0001. The scale bars in right lower corner represent 100 μm. (E–G) Illustrating the microscopic finding (200×) for identification of the intensity of glutathione peroxidase (GPx) expression (grey colour). (H) Analytical result of intensity of GPx expression, * versus other groups with different symbols (†, ‡), *p* < 0.0001. (I–K) Illustrating the microscopic finding (200×) for identification of the intensity of glutathione reductase (GR) expression (grey colour). (L) Analytical result of intensity of GR expression, * versus other groups with different symbols (†, ‡), *p* < 0.0001. The scale bars in right lower corner represent 50 μm. All statistical analyses were performed by one‐way anova, followed by Bonferroni multiple comparison post hoc test (*n* = 8 for each group). Symbols (*, †, ‡) indicate significance (at 0.05 level). MCT, monocrotaline; PTU, propylthiouracil.

To clarify whether PTU treatment could attenuate the MCT‐induced lung fibrosis and upregulation of antioxidant, histopathologic level of lung parenchyma was investigated by using the microscopic finding (Figure 8). The result of Masson's trichrome stain demonstrated that the fibrotic area of the lung parenchyma was significantly higher in MCT group than that of the SC group, and that was significantly reversed in MCT‐PTU group. On the contrary, the expression of glutathione peroxidase (Gpx) and glutathione reductase (GR) in lung parenchyma, two indicators of antioxidant enzymes, displayed an opposite pattern of lung fibrosis among the three groups. Our finding certified that PTU treatment not only suppressed the lung fibrosis but also upregulated the anti‐oxidants in setting of PAH.

### 3.11 Immunofluorescent stain for identification of specific inflammatory cells in the lung parenchyma by Day 42 after MCT treatment

**FIGURE 9:**
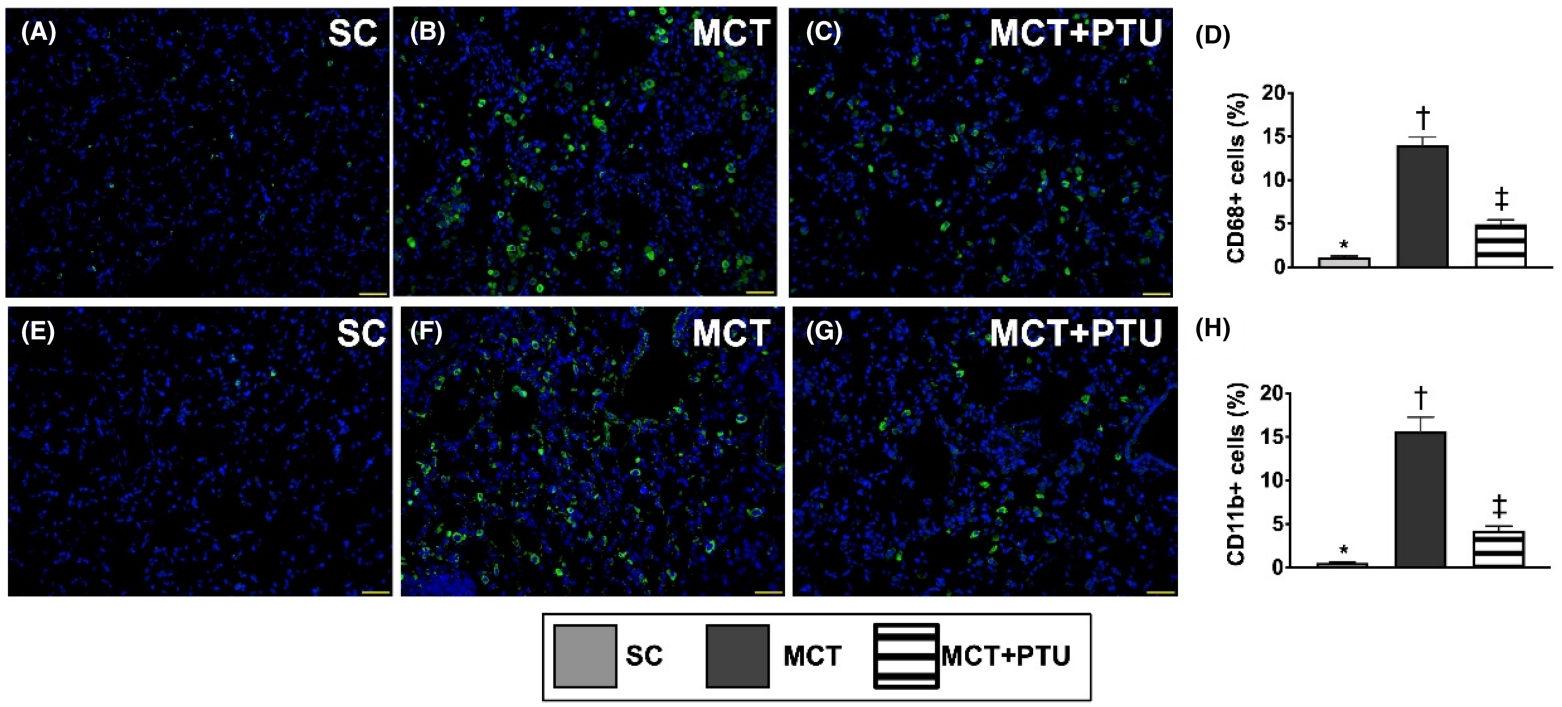
Immunofluorescent stain for identification of inflammatory cells in the lung parenchyma by Day 42 after MCT treatment. (A–C) Illustrating the immunofluorescent (IF) microscopic finding (400×) for identification of CD11b cell infiltration in the lung parenchyma (green colour). (D) Analytical result of number of CD11^+^ cells, * versus other groups with different symbols (†, ‡), *p* < 0.0001. (E–G) Illustrating the immunofluorescent (IF) microscopic finding (400×) for identification of CD68 cell infiltration in the lung parenchyma (green colour). (H) Analytical result of number of CD68^+^ cells, * versus other groups with different symbols (†, ‡), *p* < 0.0001. The scale bars in right lower corner represent 20 μm. All statistical analyses were performed by one‐way anova, followed by Bonferroni multiple comparison post hoc test (*n* = 8 for each group). Symbols (*, †, ‡) indicate significance (at 0.05 level). MCT, monocrotaline; PTU, propylthiouracil.

By using the immunofluorescent (IF) microscopic finding, we further clarified the inflammatory cell distribution in the lung parenchyma (Figure 9). The result showed that cellular expressions of CD11b and CD68, two indicators of inflammation, were significantly increased in MCT group than in SC and MCT‐PTU groups and significantly increased in MCT‐PTU group than in the SC group, suggesting that PTU therapy could attenuate inflammation in lung parenchyma of PAH. Our finding provided the evidence that PTU therapy suppressed PAH‐induced inflammatory reaction.

### 3.12 Protein expressions of pressure overload/cardiac hypertrophy, oxidative stress, DNA‐damaged and cellular jap junction biomarkers in RV myocardium by Day 42 after MCT treatment

**FIGURE 10:**
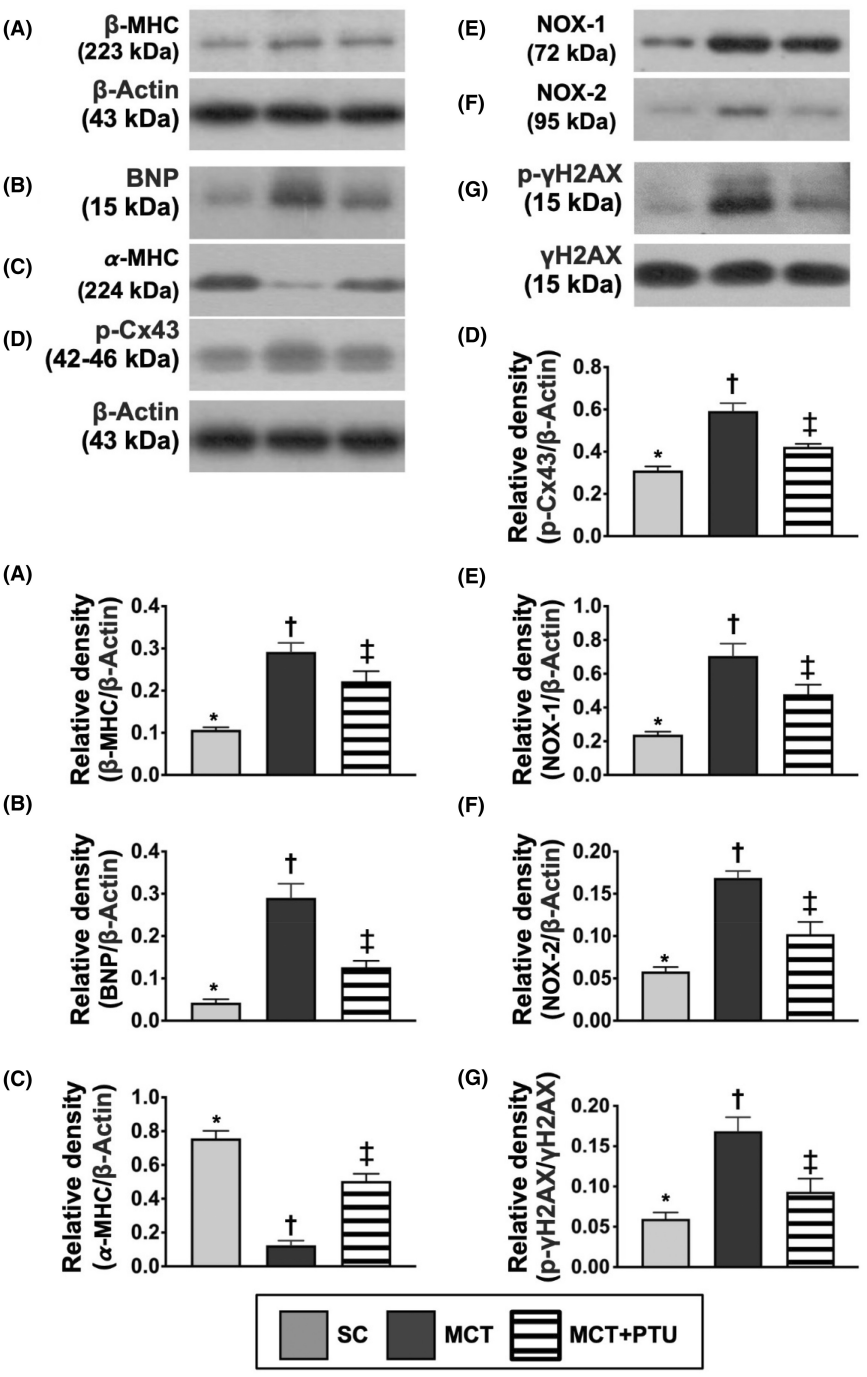
Protein expressions of pressure overload/cardiac hypertrophic, oxidative stress, DNA‐damaged and cellular jap junction biomarkers in RV myocardium by Day 42 after MCT treatment. (A) Protein expression of beta‐myosin heavy chain (ß‐MHC), * versus other groups with different symbols (†, ‡), *p* < 0.0001. (B) Protein expression of brain natriuretic peptide (BNP), * versus other groups with different symbols (†, ‡), *p* < 0.0001. (C) Protein expression of α‐MHC, * versus other groups with different symbols (†, ‡), *p* < 0.0001. (D) Protein expression of phosphorylated connexin43 (p‐Cx43), * versus other groups with different symbols (†, ‡), *p* < 0.0001. (E) Protein expression of NOX‐1, * versus other groups with different symbols (†, ‡), *p* < 0.0001. (F) Protein expression of NOX‐2. (G) Protein expression of phosphorylated (p) γ‐H2AX, * versus other groups with different symbols (†, ‡), *p* < 0.0001. All statistical analyses were performed by one‐way anova, followed by Bonferroni multiple comparison post hoc test (*n* = 6 for each group). Symbols (*, †, ‡) indicate significance (at 0.05 level). MCT, monocrotaline; PTU, propylthiouracil.

By using the RV specimen, we performed the Western blot analysis and the result demonstrated that the protein expression of ß‐MHC, an indicator of cardiac hypertrophy, the protein expression of BNP, an indicator of pressure‐overload/heart failure biomarker, protein expressions of NOX‐1 and NOX‐2, two indices of oxidative stress and γ‐H2AX, an indicator of DNA‐damaged marker, were significantly increased in MCT group than in the SC and MCT‐PTU groups and significantly increased in MCT‐PTU group than in that of the SC group (Figure 10). On the contrary, the protein expression of α‐MHC, a biomarker of reversed cardiac hypertrophy, exhibited an opposite pattern of ß‐MHC among the groups.

When we looked at the protein expression of connexin43, an indicator of jap junction for cell‐to‐cell communication, it was surprisingly identified to display an identical pattern of ß‐MHC among the groups, suggesting that it could be an intrinsic response to pressure overload stimulation.

### 3.13 Protein expressions of promoters and transcriptional regulators of endMT, and gene expression of miR‐200 in lung parenchyma by Day 42 after MCT treatment

**FIGURE 11:**
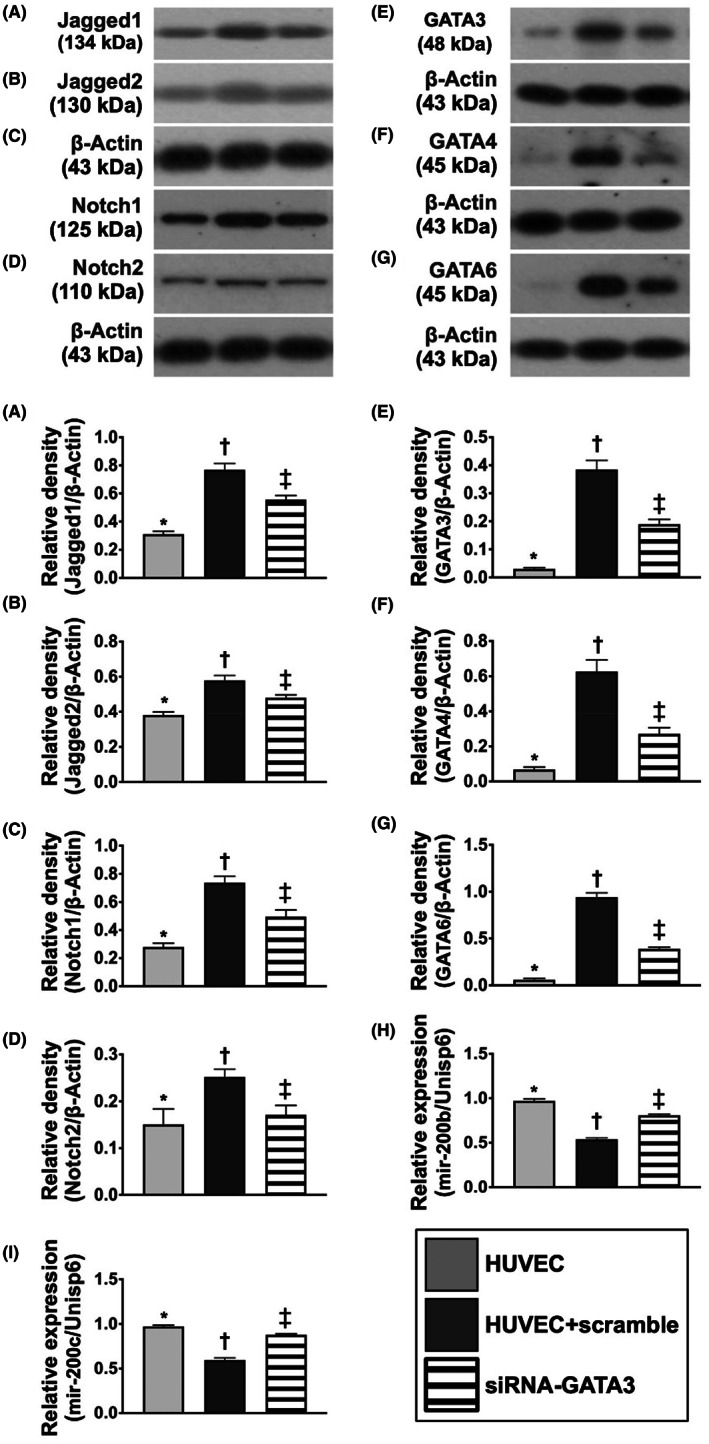
Protein expressions of promoters and transcriptional regulators of endMT and gene expression of miR‐200 in lung parenchyma by Day 42 after MCT treatment. (A) Protein expression of Jagged1, * versus other groups with different symbols (†, ‡), *p* < 0.0001. (B) Protein expression of Jagged2, * versus other groups with different symbols (†, ‡), *p* < 0.0001. (C) Protein expression of Notch1, * versus other groups with different symbols (†, ‡), *p* < 0.0001. (D) Protein expression of Notch2, * versus other groups with different symbols (†, ‡), *p* < 0.0001. (E) Protein expression of GATA3, * versus other groups with different symbols (†, ‡), *p* < 0.0001. (F) Protein expression of GATA4, * versus other groups with different symbols (†, ‡), *p* < 0.0001. (G) Protein expression of GATA6, * versus other groups with different symbols (†, ‡), *p* < 0.001. (H) Relative gene expression of miRNA‐200b‐3p, * versus other groups with different symbols (†, ‡), *p* < 0.001. (I) Relative gene expression of miRNA‐200c‐3p, * versus other groups with different symbols (†, ‡), *p* < 0.0001. All statistical analyses were performed by one‐way anova, followed by Bonferroni multiple comparison post hoc test (*n* = 6 for each group). Symbols (*, †, ‡) indicate significance (at 0.05 level). endEMT, endothelial‐mesenchymal transition; MCT, monocrotaline; PTU, propylthiouracil.

To verify whether the promoters and transcriptional regulators of endMT were upregulated in the lung of PAH rodents, Western blot analysis was utilized in the present study (Figure 11). The result demonstrated that the protein expressions of Jagged1/Jagged2 and Notch1/Notch2, four indicators of promoters, were significantly increased in MCT group than in SC and MCT‐PTU groups and significantly increased in MCT‐PTU group than in SC group. Additionally, the protein expressions of GATAs (3, 4, 6), three indices of endMT‐related transcriptional regulators, exhibited an identical pattern of the above‐mentioned promoters among the three groups. On the contrary, the gene expressions of miRNA‐200a‐3p and miRNA‐200c‐3p, two indicators of endMT‐related transcriptional repressors, displayed an opposite pattern of GATAs among the groups. Our findings highlighted miRNA‐200a‐3p and miRNA‐200c‐3p might possibly inhibit the Jagged1/Jagged2 and Notch1/Notch2 induced PAH.

### 3.14 Protein expressions of endMT biomarkers in lung parenchyma by Day 42 after MCT treatment

**FIGURE 12:**
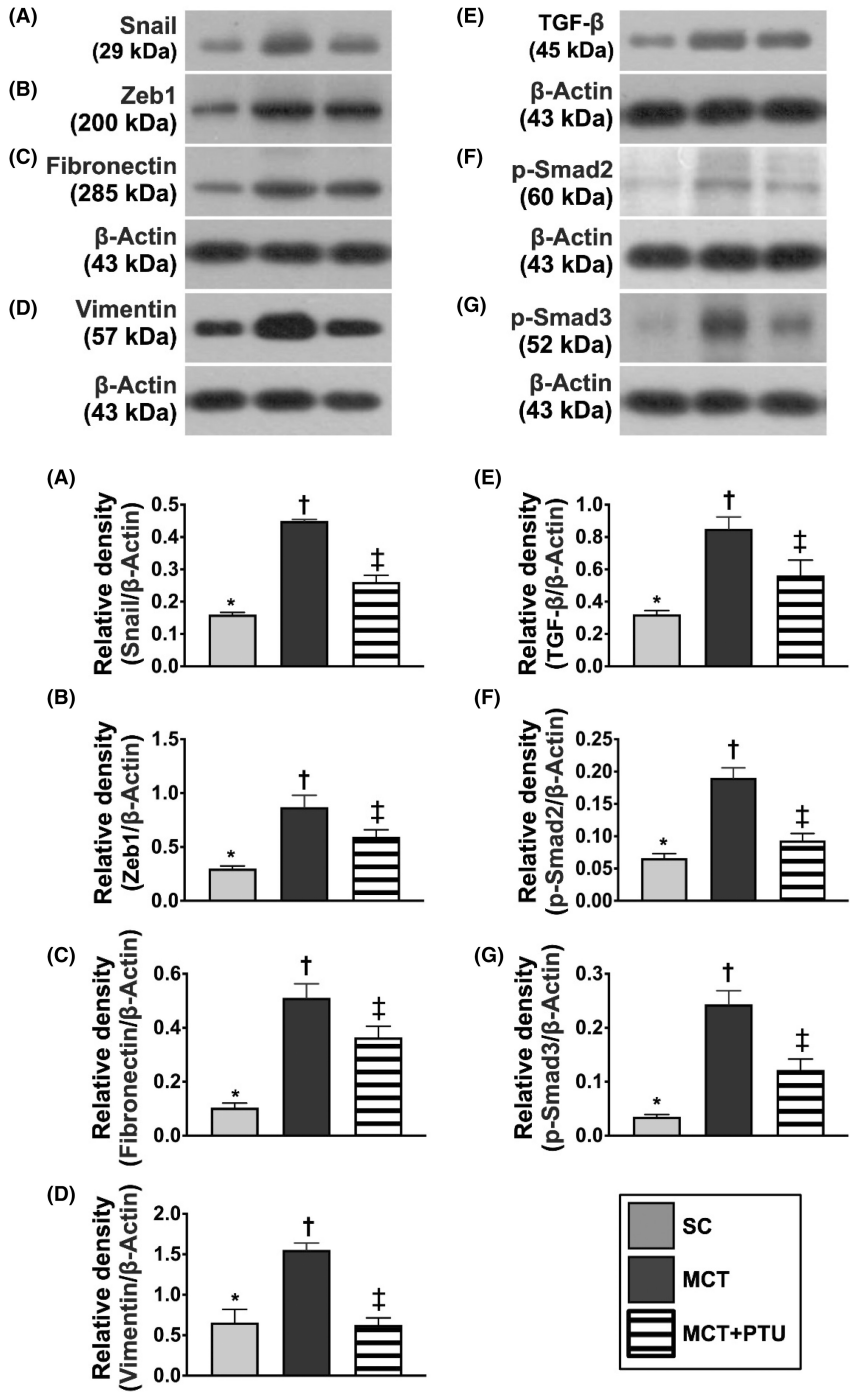
Protein expressions of endMT markers in lung parenchyma by Day 42 after MCT treatment. (A) Protein expression of Snail, * versus other groups with different symbols (†, ‡), *p* < 0.0001. (B) Protein expression of Zeb1, * versus other groups with different symbols (†, ‡), *p* < 0.0001. (C) Protein expression of Fibronectin, * versus other groups with different symbols (†, ‡), *p* < 0.0001. (D) Protein expression of Vimentin, * versus other groups with different symbols (†, ‡), *p* < 0.0001. (E) Protein expression of transforming growth factor (TGF)‐ß, * versus other groups with different symbols (†, ‡), *p* < 0.0001. (F) Protein expression of phosphorylated (p)‐Smad2, * versus other groups with different symbols (†, ‡), *p* < 0.0001. (G) Protein expression of p‐Smad3, * versus other groups with different symbols (†, ‡), *p* < 0.0001. All statistical analyses were performed by one‐way anova, followed by Bonferroni multiple comparison post hoc test (*n* = 6 for each group). Symbols (*, †, ‡) indicate significance (at 0.05 level). endEMT, endothelial‐mesenchymal transition; MCT, monocrotaline; PTU, propylthiouracil.

To clarify whether the PTU therapy effectively attenuated the expression of endMT biomarkers in PHA rat, Western blot analysis of the lung tissue was performed again (Figure 12). The result demonstrated that the protein expressions of Snail, Zeb1, fibronectin, Vimentin, TGF‐ß, p‐Smad2 and p‐Smad3, seven indices of endMT biomarkers, were significantly increased in MCT group than in the SC group, and were significantly reversed in MCT‐PTU group, suggesting PTU therapy may be potential for the PAH patients.

## 4 DISCUSSION

This study which investigated the role of Jaggeds‐Notches‐GATATs‐endMT signalling pathway on the initiation and propagation of MCT‐induced PAH in rodent and the therapeutic impact of PTU on this disease entity yielded several striking preclinical implications. First, we successfully created an MCT‐induced PAH model in rodent and found that the endMT biomarkers were substantially upregulated in setting of PAH. Second, in vitro and in vivo studies strongly demonstrated that both MCT and TGF‐ß played crucial roles on upregulation of endMT‐related promoters. Third, silenced *JAG2* gene (i.e. endMT promoter) and *GATA3* gene (i.e. endMT transcriptional regulator) remarkably suppressed the expression of endMT biomarkers, suggesting that the *JAG2‐GATA3* signalling was essential for the endMT initiation and propagation of endMT and PAH. Finally, the present study demonstrated that all of the endMT promoters/endMT transcriptional regulators and endMT biomarkers in HUVECs and lung tissues were significantly suppressed by PTU treatment.

The distinctively histopathological feature of pulmonary artery in setting of PAH has been well recognized as endlessly increasing in the intimal, medial and adventitial layers, resulting in excessive pulmonary vascular remodelling and sustained pulmonary vasoconstriction, as a consequence of PAH and right‐side heart failure.^50^

It is well investigated that the signal transduction in Notch pathway is elicited by binding of Notch receptors (i.e. Notch1 to 4) with ligands (i.e. Jagged1, Jagged2, Delta‐like, Delta‐like3 and Delta‐like4).^51^, ^52^ Additionally, Notch signalling has been identified to not only participate in proliferation, invasion and metastasis of cancer cells/cancer stem‐like cells^10^, ^11^ but also be particularly important in vascular remodelling by promoting proliferation of endothelial cells and smooth muscle cell recruitment in setting of PAH.^50^, ^53^, ^54^

One important finding in the present study was that the protein expressions of Notch1/Notch2 and Jagged1/Jagged2 were significantly upregulated in HUVECs undergoing the TGF‐ß or MCT treatment. Additionally, these parameters were also identified to be markedly increased in lung parenchyma of MCT‐induced PAH rats. Furthermore, the protein expressions of the endMT markers were substantially increased in HUVECs‐treated by TGF‐ß or MCT and lung tissue of PAH rats. On the contrary, when looked at the in vitro study, we also identified that silenced *JAG2* gene in HUVECs significantly suppressed the protein expressions of the endMT markers. Our findings, in addition to being consistent with the findings of the previous studies,^50^, ^51^, ^52^, ^53^, ^54^ suggested that Notches‐Jaggeds signalling would serve as a cardinal driver of PAH pathogenesis.

Previous study has revealed that GATA factors regulated by Jagged2 which has been identified to essentially promote metastasis by enhancing the expression of GATA‐binding factors.^8^ Another important finding in the present study was that the protein expressions of GATAs were significantly enhanced in TGF‐ß treated and in MCT treated HUVECs as well as notably increased in lung tissues of PAH rodents. Accordingly, our findings, in addition to being comparable with the findings of the previous study,^8^ highlighted that the high level of GATAs expression was crucial for upregulation of endMT expressions in PAH. In fact, the role of GATAs in the development of PAH is still currently unclear. Interestingly, one previous study has shown that a critical GATA‐6 deficiency played a critical role in the development and disease progression in PAH.^55^ Another previous study has also revealed that the levels of *GATA‐6* mRNA and protein were markedly decreased in those of pneumonectomy and MCT‐treated PAH rats.^56^ In contrast to these studies,^55^, ^56^ Suzuki et al previously demonstrated that GATA‐4 activation crucially regulated pulmonary arterial smooth muscle cell proliferation, suggesting targeting at the GATA‐4 activation could be a therapeutic potential for preventing the PAH.^28^ In this way, our finding was consistent with the finding of the previous study.^28^ We remain uncertain regarding why the protein expressions of GATAs were remarkably increased not only in the in vitro study (i.e. HUVECs treated by TGF‐ß or by MCT) but also in the lung parenchyma of PAH rats. Perhaps, one main reason could be due to an intrinsic response of the HUVECs and the cells in lung parenchyma to the MCT stimulation or hypoxia/elevated RVSBP, that is an index of PA systolic blood pressure.

The miR‐200 family are universally accepted to target at the transcriptional repressors, resulting in inhibiting the EMT and cancer cell metastasis.^10^, ^25^ Surprisingly, while an association between tumour metastasis along with upregulation of EMT and downregulation of miR‐200 has been extensively investigated^8^, ^28^, ^57^ link between miR‐200 and PAH has not yet been reported. An essential finding in the present study was that the miR‐200a‐3p and miR‐200c‐3P were markedly suppressed by MCT treatment (i.e. in vitro study) and in lung parenchyma of PAH rat. Accordingly, our findings, in addition to extending of the previous studies,^8^, ^28^, ^57^ highlight that this finding could possess novelty, and therefore, would provide a more complete jigsaw puzzle on the understanding of PAH mechanism.

PAH always causes the RV pressure overload, RV hypertrophy and finally, right‐side heart failure.^1^, ^2^, ^3^, ^5^ A principal finding in the present study was that the parameters of RV weight, lung weight and the RVSBP were significantly increased in MCT group than in SC control, whereas the SaO_2_ exhibited an opposite pattern between these two groups. Additionally, protein expressions of pressure overload, hypertrophic and oxidative biomarkers remarkably increased in MCT animals than in the SC animals. Our findings were consistent with the findings of those previous studies,^1^, ^2^, ^3^, ^5^ suggesting that we had successfully created a reliable animal model of PAH for the study.

Interestingly, our previous study has proved that PTU therapy significantly attenuated MCT‐induced PAH, RVSBP, RV hypertrophy and lung injury in rodent.^45^ However, the limitation of our study^45^ was that the underlying mechanisms of how the PTU therapy could ameliorate the above‐mentioned parameters and the PAH were not clearly verified. Our present study also demonstrated that the PTU therapy effectively improved the MCT‐induced PAH and protected the right ventricle and lung organ against the PAH‐induced injury. In this way, our finding corroborated with the finding of our previous study.^45^ Of distinctively important finding in the present study was that the underlying mechanism of PTU therapy attenuated the MCT‐induced PAH was clearly clarified due to inhibiting the endMT processing up‐regulators (i.e. Jagged1/Jagged2 and Notch1/Notch2) and endMT transcriptional regulators (i.e. GATAs [3, 4, 6]), and upregulating the miR‐200a‐3p/miR‐200c‐3p (i.e. transcriptional repressor), resulting in suppression of the initiation and propagation of endMT and PAH.

### 4.1 Study limitation

**FIGURE 13:**
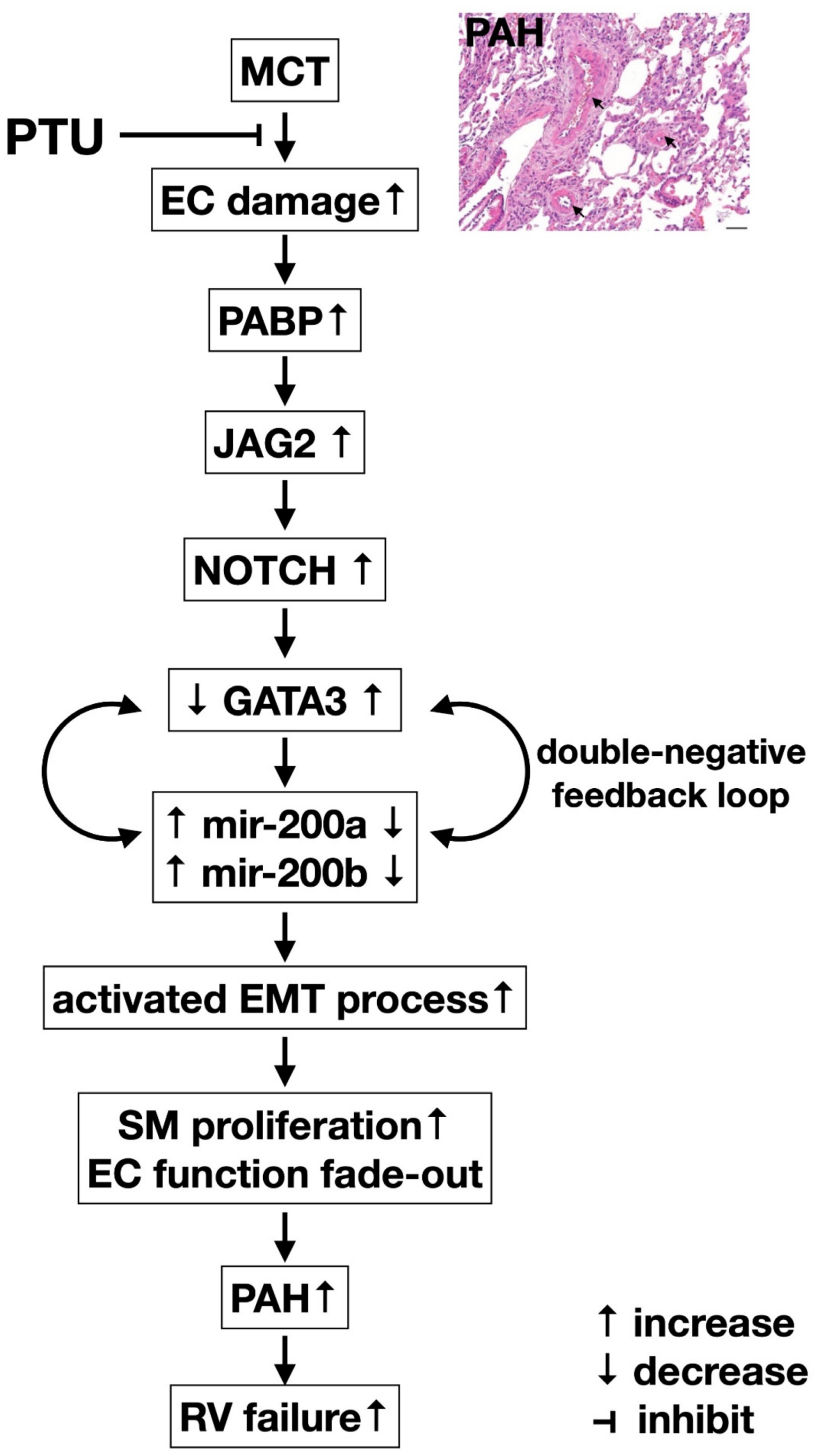
Schematically illustrating the underlying mechanism of monocrotaline‐induced endothelial damage mimicking the clinical setting of pulmonary arterial hypertension (PAH) and the therapeutic impact of propylthiouracil on against the initiation and propagation of PAH. EC, endothelial cell; endMT, endothelial‐mesenchymal transition; PABP, pulmonary arterial blood pressure; RV, right ventricular; SM, smooth muscle. Black arrows indicated medium layer thickness after monocrotaline (MCT) treatment.

This study has limitations. First, the dosage of PTU utilized in the present study was merely based on our previous report.^45^ Accordingly, this study did not verify what was the optimal dose for offering the best impact on inhibiting the PAH. Second, although previous studies have demonstrated that the protective effect of PTU on inhibiting the atherosclerosis, smooth muscle cell proliferation and PAH was independent of its hypothyroid effect^39^, ^40^, ^41^, ^42^, ^43^, ^44^, ^45^ and no PTU‐related hypothyroidism side effect was observed by previous studies,^41^, ^45^ the present study did not measure the circulating level of TSH, T3 or free T4 in the rodent. However, we did not find any PTU‐related hypothyroidism side effect in the animals. Third, this study just utilized the HUVECs rather than to utilize the rat circulatory‐derived endothelial cells or primary endothelial cells from PAH patient to conduct the endEMT, suggesting that its justifiability might not be enough. Finally, although extensive works were done in the present study, the precise mechanism of PAH might be more complicated that was out of the scope of this study. Based on the results of our study, we schematically illustrated the underlying mechanism of how the PTU therapy suppressed the development of MCT‐induced PAH in rodent in Figure 13.

In conclusion, the result of this study demonstrated that Notch‐Jagged‐GATA signalling participated in regulating the endMT expression and suppressing the miR‐200 family in setting of PAH were significantly reversed by PTU therapy.

## AUTHOR CONTRIBUTIONS

**Kun‐Chen Lin:** Formal analysis (equal); funding acquisition (equal); investigation (equal); methodology (equal). **Jui‐Ning Yeh:** Data curation (equal); formal analysis (equal); investigation (equal). **Pei‐Lin Shao:** Data curation (equal); formal analysis (equal); investigation (equal); writing – review and editing (equal). **John Y. Chiang:** Writing – review and editing (equal). **Pei‐Hsun Sung:** Data curation (equal); formal analysis (equal); investigation (equal); methodology (equal); writing – review and editing (equal). **Chi‐Ruei Huang:** Data curation (equal); formal analysis (equal); investigation (equal). **Yi‐Ling Chen:** Data curation (equal); formal analysis (equal); investigation (equal). **Hon‐Kan Yip:** Methodology (equal); supervision (equal); writing – original draft (equal). **Jun Guo:** Funding acquisition (equal); methodology (equal); supervision (equal); writing – review and editing (equal).

## FUNDING INFORMATION

This study was supported by a program grant from Chang Gung Memorial Hospital, Chang Gung University, Taiwan [CMRPG8K1051] and Guangzhou Science and technology planning project, China (202103000010).

## CONFLICT OF INTEREST STATEMENT

The authors declare no conflict of interest.

## Supporting information


Figure S1–S3

## Data Availability

The datasets of present study can be available from the corresponding author upon request.

## References

[1] Pietra GG , Edwards WD , Kay JM , et al. Histopathology of primary pulmonary hypertension. A qualitative and quantitative study of pulmonary blood vessels from 58 patients in the National Heart, Lung, and Blood Institute, primary pulmonary hypertension registry. Circulation. 1989;80:1198‐1206. 2805258 10.1161/01.cir.80.5.1198

[2] D'Alonzo GE , Barst RJ , Ayres SM , et al. Survival in patients with primary pulmonary hypertension. Results from a national prospective registry. Ann Intern Med. 1991;115:343‐349. 1863023 10.7326/0003-4819-115-5-343

[3] Rubin LJ . Primary pulmonary hypertension. N Engl J Med. 1997;336:111‐117. 8988890 10.1056/NEJM199701093360207

[4] Gaine SP , Rubin LJ . Primary pulmonary hypertension. Lancet. 1998;352:719‐725. 9729004 10.1016/S0140-6736(98)02111-4

[5] Runo JR , Loyd JE . Primary pulmonary hypertension. Lancet. 2003;361:1533‐1544. 12737878 10.1016/S0140-6736(03)13167-4

[6] Rubin LJ , Badesch DB . Evaluation and management of the patient with pulmonary arterial hypertension. Ann Intern Med. 2005;143:282‐292. 16103472 10.7326/0003-4819-143-4-200508160-00009

[7] Archer SL , Michelakis ED . Phosphodiesterase type 5 inhibitors for pulmonary arterial hypertension. N Engl J Med. 2009;361:1864‐1871. 19890129 10.1056/NEJMct0904473

[8] Yang Y , Ahn YH , Gibbons DL , et al. The Notch ligand Jagged2 promotes lung adenocarcinoma metastasis through a miR‐200‐dependent pathway in mice. J Clin Invest. 2011;121:1373‐1385. 21403400 10.1172/JCI42579 PMC3069760

[9] Hu Y , Su H , Li X , et al. The NOTCH ligand JAGGED2 promotes pancreatic cancer metastasis independent of NOTCH signaling activation. Mol Cancer Ther. 2015;14:289‐297. 25351917 10.1158/1535-7163.MCT-14-0501

[10] Xing F , Okuda H , Watabe M , et al. Hypoxia‐induced Jagged2 promotes breast cancer metastasis and self‐renewal of cancer stem‐like cells. Oncogene. 2011;30:4075‐4086. 21499308 10.1038/onc.2011.122 PMC3145824

[11] Mullendore ME , Koorstra JB , Li YM , et al. Ligand‐dependent Notch signaling is involved in tumor initiation and tumor maintenance in pancreatic cancer. Clin Cancer Res. 2009;15:2291‐2301. 19258443 10.1158/1078-0432.CCR-08-2004 PMC2711441

[12] Li W , Liu M , Feng Y , et al. High expression of Notch ligand Jagged2 is associated with the metastasis and recurrence in urothelial carcinoma of bladder. Int J Clin Exp Pathol. 2013;6:2430‐2440. 24228105 PMC3816812

[13] Fang TC , Yashiro‐Ohtani Y , Del Bianco C , Knoblock DM , Blacklow SC , Pear WS . Notch directly regulates Gata3 expression during T helper 2 cell differentiation. Immunity. 2007;27:100‐110. 17658278 10.1016/j.immuni.2007.04.018 PMC2801546

[14] Zhang C , Ye X , Zhang H , Ding M , Deng H . GATA factors induce mouse embryonic stem cell differentiation toward extraembryonic endoderm. Stem Cells Dev. 2007;16:605‐613. 17784834 10.1089/scd.2006.0077

[15] Hoey T , Yen WC , Axelrod F , et al. DLL4 blockade inhibits tumor growth and reduces tumor‐initiating cell frequency. Cell Stem Cell. 2009;5:168‐177. 19664991 10.1016/j.stem.2009.05.019

[16] Lentjes MH , Niessen HE , Akiyama Y , de Bruine AP , Melotte V , van Engeland M . The emerging role of GATA transcription factors in development and disease. Expert Rev Mol Med. 2016;18:e3. 26953528 10.1017/erm.2016.2 PMC4836206

[17] Chou J , Provot S , Werb Z . GATA3 in development and cancer differentiation: cells GATA have it! J Cell Physiol. 2010;222:42‐49. 19798694 10.1002/jcp.21943 PMC2915440

[18] Agarwal S , Loder S , Cholok D , et al. Local and circulating endothelial cells undergo endothelial to mesenchymal transition (EndMT) in response to musculoskeletal injury. Sci Rep. 2016;6:32514. 27616463 10.1038/srep32514 PMC5018841

[19] Suzuki T , Carrier EJ , Talati MH , et al. Isolation and characterization of endothelial‐to‐mesenchymal transition cells in pulmonary arterial hypertension. Am J Physiol Lung Cell Mol Physiol. 2018;314:L118‐L126. 28935639 10.1152/ajplung.00296.2017 PMC5866427

[20] Yue Y , Zhang Z , Zhang L , Chen S , Guo Y , Hong Y . miR‐143 and miR‐145 promote hypoxia‐induced proliferation and migration of pulmonary arterial smooth muscle cells through regulating ABCA1 expression. Cardiovasc Pathol. 2018;37:15‐25. 30195228 10.1016/j.carpath.2018.08.003

[21] Liu T , Zou XZ , Huang N , et al. miR‐27a promotes endothelial‐mesenchymal transition in hypoxia‐induced pulmonary arterial hypertension by suppressing BMP signaling. Life Sci. 2019;227:64‐73. 31004656 10.1016/j.lfs.2019.04.038

[22] Liu T , Zou XZ , Huang N , et al. Down‐regulation of miR‐204 attenuates endothelial‐mesenchymal transition by enhancing autophagy in hypoxia‐induced pulmonary hypertension. Eur J Pharmacol. 2019;863:172673. 31542480 10.1016/j.ejphar.2019.172673

[23] Piera‐Velazquez S , Jimenez SA . Endothelial to mesenchymal transition: role in physiology and in the pathogenesis of human diseases. Physiol Rev. 2019;99:1281‐1324. 30864875 10.1152/physrev.00021.2018 PMC6734087

[24] Eger A , Aigner K , Sonderegger S , et al. DeltaEF1 is a transcriptional repressor of E‐cadherin and regulates epithelial plasticity in breast cancer cells. Oncogene. 2005;24:2375‐2385. 15674322 10.1038/sj.onc.1208429

[25] Aigner K , Dampier B , Descovich L , et al. The transcription factor ZEB1 (deltaEF1) promotes tumour cell dedifferentiation by repressing master regulators of epithelial polarity. Oncogene. 2007;26:6979‐6988. 17486063 10.1038/sj.onc.1210508 PMC2899859

[26] Wellner U , Schubert J , Burk UC , et al. The EMT‐activator ZEB1 promotes tumorigenicity by repressing stemness‐inhibiting microRNAs. Nat Cell Biol. 2009;11:1487‐1495. 19935649 10.1038/ncb1998

[27] Burk U , Schubert J , Wellner U , et al. A reciprocal repression between ZEB1 and members of the miR‐200 family promotes EMT and invasion in cancer cells. EMBO Rep. 2008;9:582‐589. 18483486 10.1038/embor.2008.74 PMC2396950

[28] Gregory PA , Bert AG , Paterson EL , et al. The miR‐200 family and miR‐205 regulate epithelial to mesenchymal transition by targeting ZEB1 and SIP1. Nat Cell Biol. 2008;10:593‐601. 18376396 10.1038/ncb1722

[29] Park SM , Gaur AB , Lengyel E , Peter ME . The miR‐200 family determines the epithelial phenotype of cancer cells by targeting the E‐cadherin repressors ZEB1 and ZEB2. Genes Dev. 2008;22:894‐907. 18381893 10.1101/gad.1640608 PMC2279201

[30] Gibbons DL , Lin W , Creighton CJ , et al. Contextual extracellular cues promote tumor cell EMT and metastasis by regulating miR‐200 family expression. Genes Dev. 2009;23:2140‐2151. 19759262 10.1101/gad.1820209 PMC2751985

[31] Park A , Liu L , Wong C , Suzuki YJ . Pulmonary hypertension activates GATA‐4 in the right heart: role of CBF/NF‐Y in the regulation of Gata4 gene transcription. C27 Right Ventricular Function and Exercise. American Thoracic Society; 2009:A4140.

[32] Ryan K , Shah N , Holland S , et al. Resolution of Pulmonary Arterial Hypertension (PAH) in a Patient with GATA‐2 Deficiency Following Bone Marrow Transplant (BMT). Am J Respir Crit Care Med. 2019;199:1.30183328

[33] Fan Y , Gu X , Zhang J , et al. TWIST1 drives smooth muscle cell proliferation in pulmonary hypertension via loss of GATA‐6 and BMPR2. Am J Respir Crit Care Med. 2020;202:1283‐1296. 32692930 10.1164/rccm.201909-1884OC

[34] Suzuki YJ , Day RM , Tan CC , et al. Activation of GATA‐4 by serotonin in pulmonary artery smooth muscle cells. J Biol Chem. 2003;278:17525‐17531. 12615926 10.1074/jbc.M210465200

[35] Isobe S , Kataoka M , Endo J , et al. Endothelial‐mesenchymal transition drives expression of CD44 variant and xCT in pulmonary hypertension. Am J Respir Cell Mol Biol. 2019;61:367‐379. 30897333 10.1165/rcmb.2018-0231OC

[36] Zhang H , Lin Y , Ma Y , Zhang J , Wang C , Zhang H . Protective effect of hydrogen sulfide on monocrotaline‐induced pulmonary arterial hypertension via inhibition of the endothelial mesenchymal transition. Int J Mol Med. 2019;44:2091‐2102. 31573044 10.3892/ijmm.2019.4359 PMC6844600

[37] Yamamura H , Yamamura A , Ko EA , et al. Activation of Notch signaling by short‐term treatment with Jagged‐1 enhances store‐operated Ca(2+) entry in human pulmonary arterial smooth muscle cells. Am J Physiol Cell Physiol. 2014;306:C871‐C878. 24573085 10.1152/ajpcell.00221.2013 PMC4010807

[38] Zhang Y , Hernandez M , Gower J , et al. JAGGED‐NOTCH3 signaling in vascular remodeling in pulmonary arterial hypertension. Sci Transl Med. 2022;14:eabl5471. 35507674 10.1126/scitranslmed.abl5471

[39] Hicks M , Wong LS , Day RO . Antioxidant activity of propylthiouracil. Biochem Pharmacol. 1992;43:439‐444. 1311579 10.1016/0006-2952(92)90561-v

[40] Grieve DJ , Fletcher S , Pitsillides AA , Botham KM , Elliott J . Effects of oral propylthiouracil treatment on nitric oxide production in rat aorta. Br J Pharmacol. 1999;127:1‐8. 10369449 10.1038/sj.bjp.0702501 PMC1565988

[41] Chen WJ , Ho WJ , Chang GJ , et al. Propylthiouracil, independent of its antithyroid effect, produces endothelium‐dependent vasodilatation through induction of nitric oxide bioactivity. Atherosclerosis. 2008;196:383‐390. 17178124 10.1016/j.atherosclerosis.2006.11.018

[42] Chen WJ , Lin KH , Lai YJ , Yang SH , Pang JH . Protective effect of propylthiouracil independent of its hypothyroid effect on atherogenesis in cholesterol‐fed rabbits: PTEN induction and inhibition of vascular smooth muscle cell proliferation and migration. Circulation. 2004;110:1313‐1319. 15326060 10.1161/01.CIR.0000140764.15398.F3

[43] Chen WJ , Pang JH , Lin KH , Yang SH . Propylthiouracil, independent of its antithyroid effect, decreases VSMC collagen expression. Basic Res Cardiol. 2009;104:60‐68. 18820824 10.1007/s00395-008-0746-8

[44] Lin PY , Lee FY , Wallace CG , et al. The therapeutic effect of rosuvastatin and propylthiouracil on ameliorating high‐cholesterol diet‐induced rabbit aortic atherosclerosis and stiffness. Int J Cardiol. 2017;227:938‐949. 27932239 10.1016/j.ijcard.2016.09.040

[45] Sun CK , Yuen CM , Kao YH , et al. Propylthiouracil attenuates monocrotaline‐induced pulmonary arterial hypertension in rats. Circ J. 2009;73:1722‐1730. 19602776 10.1253/circj.cj-09-0074

[46] Huang TH , Chung SY , Chua S , et al. Effect of early administration of lower dose versus high dose of fresh mitochondria on reducing monocrotaline‐induced pulmonary artery hypertension in rat. Am J Transl Res. 2016;8:5151‐5168. 28077992 PMC5209472

[47] Sun CK , Lin YC , Yuen CM , et al. Enhanced protection against pulmonary hypertension with sildenafil and endothelial progenitor cell in rats. Int J Cardiol. 2012;162:45‐58. 21620490 10.1016/j.ijcard.2011.05.002

[48] Sun CK , Leu S , Hsu SY , et al. Mixed serum‐deprived and normal adipose‐derived mesenchymal stem cells against acute lung ischemia‐reperfusion injury in rats. Am J Transl Res. 2015;7:209‐231. 25901192 PMC4399087

[49] Yen CH , Leu S , Lin YC , et al. Sildenafil limits monocrotaline‐induced pulmonary hypertension in rats through suppression of pulmonary vascular remodeling. J Cardiovasc Pharmacol. 2010;55:574‐584. 20224427 10.1097/FJC.0b013e3181d9f5f4

[50] Babicheva A , Yuan JX . Endothelial Notch1 in pulmonary hypertension. Circ Res. 2019;124:176‐179. 30653427 10.1161/CIRCRESAHA.118.314496 PMC6368966

[51] Bray SJ . Notch signalling in context. Nat Rev Mol Cell Biol. 2016;17:722‐735. 27507209 10.1038/nrm.2016.94

[52] Zong D , Ouyang R , Li J , Chen Y , Chen P . Notch signaling in lung diseases: focus on Notch1 and Notch3. Ther Adv Respir Dis. 2016;10:468‐484. 27378579 10.1177/1753465816654873 PMC5933616

[53] Li X , Zhang X , Leathers R , et al. Notch3 signaling promotes the development of pulmonary arterial hypertension. Nat Med. 2009;15:1289‐1297. 19855400 10.1038/nm.2021 PMC2780347

[54] Smith KA , Voiriot G , Tang H , et al. Notch activation of Ca(2+) signaling in the development of hypoxic pulmonary vasoconstriction and pulmonary hypertension. Am J Respir Cell Mol Biol. 2015;53:355‐367. 25569851 10.1165/rcmb.2014-0235OC PMC4566064

[55] Ghatnekar A , Chrobak I , Reese C , et al. Endothelial GATA‐6 deficiency promotes pulmonary arterial hypertension. Am J Pathol. 2013;182:2391‐2406. 23583651 10.1016/j.ajpath.2013.02.039 PMC3668018

[56] Liu B , Wang XQ , Yu L , Zhou TF , Wang XM , Liu HM . Simvastatin restores down‐regulated GATA‐6 expression in pulmonary hypertensive rats. Exp Lung Res. 2009;35:411‐426. 19842842 10.1080/01902140902736819 PMC2707177

[57] Zhang J , Shao N , Yang X , Xie C , Shi Y , Lin Y . Interleukin‐8 promotes epithelial‐to‐mesenchymal transition via downregulation of Mir‐200 family in breast cancer cells. Technol Cancer Res Treat. 2020;19:1533033820979672. 33280520 10.1177/1533033820979672 PMC7724258

