# Supplementary figures and images for: Jagged/Notch proteins promote endothelial‐mesenchymal transition‐mediated pulmonary arterial hypertension via upregulation of the expression of GATAs
Source: J Cell Mol Med. 2023 Mar 20;27(8):1110–30. doi: 10.1111/jcmm.17723 (PMC10098301; doi:10.1111/jcmm.17723)

Supplementary figure 1


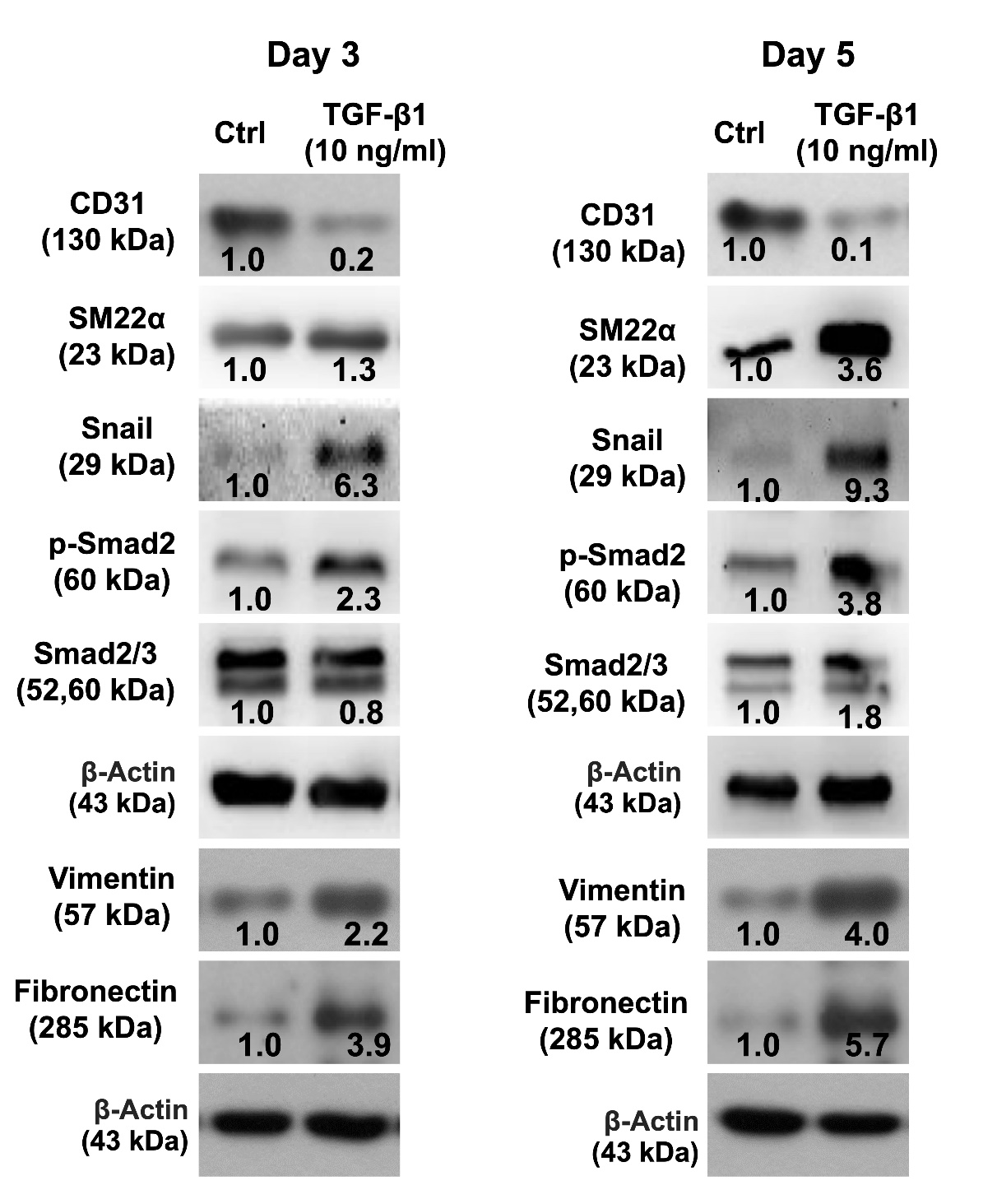


Supplementary figure 2


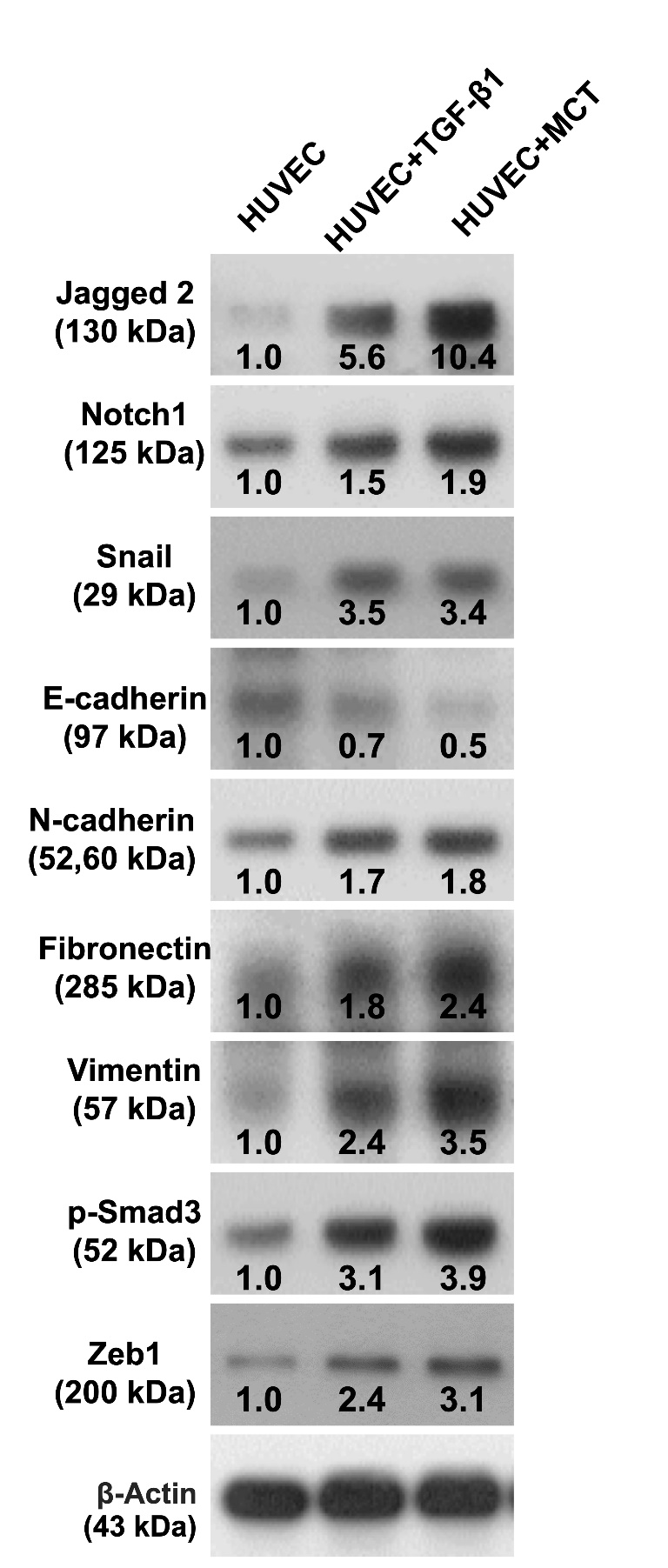


Supplementary figure 3


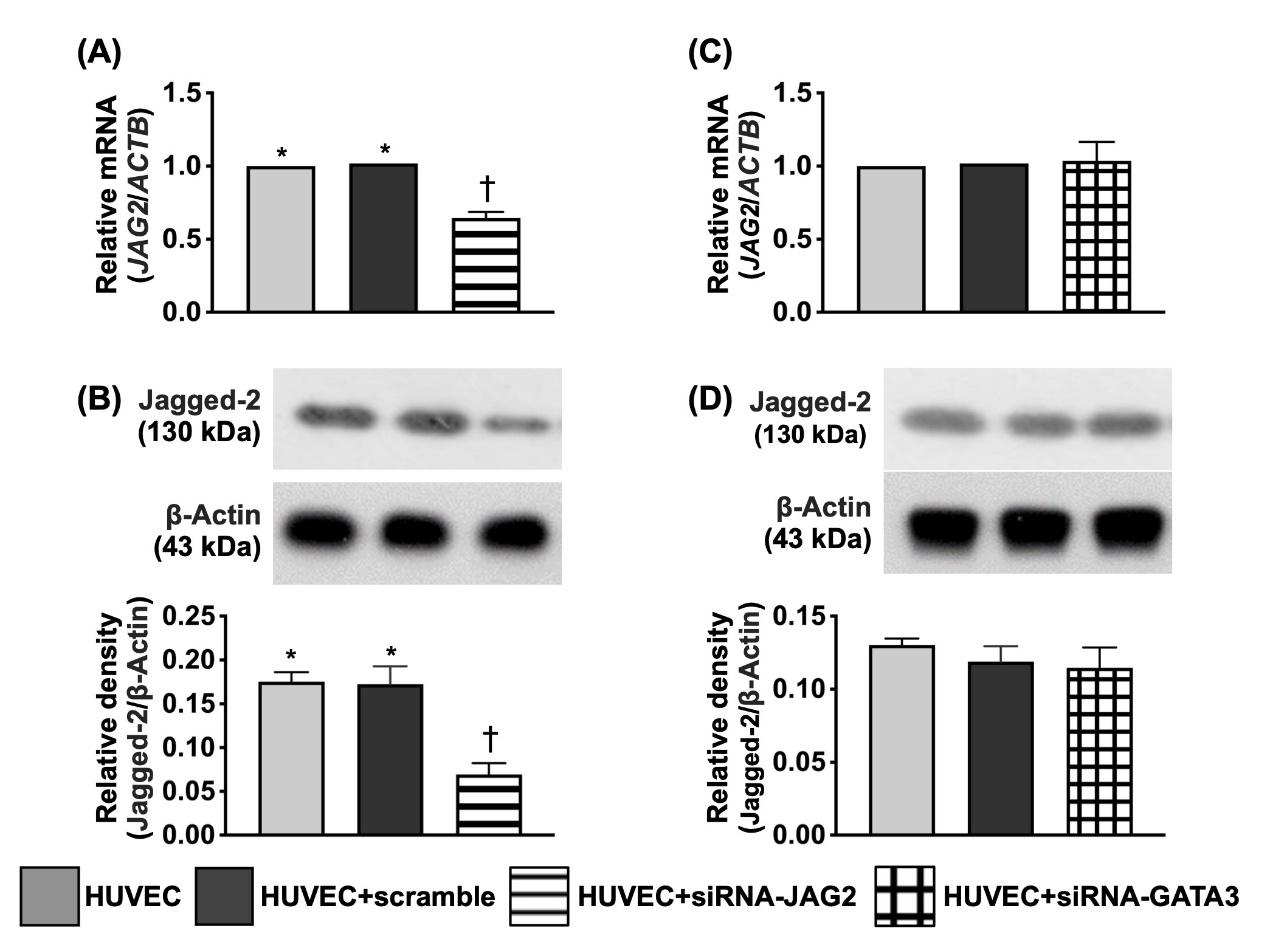

Supplement: Supplementary file 1 — Figure S1–S3 [file JCMM-27-1110-s001.docx]
